# Mouse sarcopenia model reveals sex- and age-specific differences in phenotypic and molecular characteristics

**DOI:** 10.1172/JCI172890

**Published:** 2024-06-11

**Authors:** Haiming L. Kerr, Kora Krumm, Barbara Anderson, Anthony Christiani, Lena Strait, Theresa Li, Brynn Irwin, Siyi Jiang, Artur Rybachok, Amanda Chen, Elizabeth Dacek, Lucas Caeiro, Gennifer E. Merrihew, James W. MacDonald, Theo K. Bammler, Michael J. MacCoss, Jose M. Garcia

**Affiliations:** 1 Geriatric Research, Education and Clinical Center, Veterans Affairs Puget Sound Health Care System, Seattle, Washington, USA.; 2 Division of Gerontology and Geriatric Medicine, Department of Medicine, University of Washington School of Medicine, Seattle, Washington, USA.; 3 Department of Genome Sciences, and; 4 Department of Environmental and Occupational Health Sciences, University of Washington, Seattle, Washington, USA.

**Keywords:** Aging, Muscle biology, Mitochondria, Mouse models, Skeletal muscle

## Abstract

Our study was to characterize sarcopenia in C57BL/6J mice using a clinically relevant definition to investigate the underlying molecular mechanisms. Aged male (23–32 months old) and female (27–28 months old) C57BL/6J mice were classified as non-, probable-, or sarcopenic based on assessments of grip strength, muscle mass, and treadmill running time, using 2 SDs below the mean of their young counterparts as cutoff points. A 9%–22% prevalence of sarcopenia was identified in 23–26 month-old male mice, with more severe age-related declines in muscle function than mass. Females aged 27–28 months showed fewer sarcopenic but more probable cases compared with the males. As sarcopenia progressed, a decrease in muscle contractility and a trend toward lower type IIB fiber size were observed in males. Mitochondrial biogenesis, oxidative capacity, and AMPK-autophagy signaling decreased as sarcopenia progressed in males, with pathways linked to mitochondrial metabolism positively correlated with muscle mass. No age- or sarcopenia-related changes were observed in mitochondrial biogenesis, OXPHOS complexes, AMPK signaling, mitophagy, or atrogenes in females. Our results highlight the different trajectories of age-related declines in muscle mass and function, providing insights into sex-dependent molecular changes associated with sarcopenia progression, which may inform the future development of novel therapeutic interventions.

## Abstract 2

A mouse model facilitates a clinically relevant definition for sarcopenia and reveals age-related and molecular changes underlying decreased muscle mass and function in both sexes.

## Graphical abstract

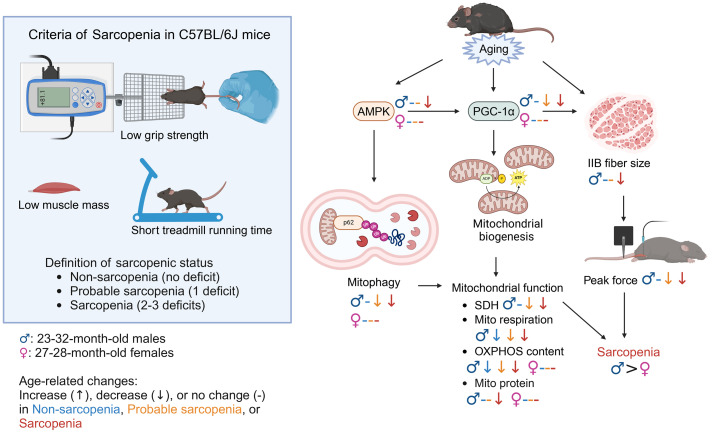

## Introduction

Sarcopenia, the loss of muscle mass and function due to aging, affects 25%–45% of older adults in the United States (1) and is associated with increased incidence of falls and injuries, cardiac disease, respiratory disease, and cognitive impairment, and is closely linked to frailty, poor quality of life, and increased mortality (2, 3). Currently, exercise is the only recognized treatment for sarcopenia (4, 5), while nutritional supplementation (6, 7) and off-label pharmacological interventions (8, 9) have shown limited efficacy in improving muscle strength and physical function (10). Therefore, sarcopenia remains an unmet clinical need. Several cellular and molecular mechanisms have been proposed as important regulators of muscle mass during aging, including fiber type transformation (11, 12), imbalance in protein homeostasis (13, 14), and mitochondrial dysfunction (15). However, alterations in these mechanisms are not consistent across studies, and their relative contribution to muscle function loss is still unclear.

Preclinical studies are essential tools to understand the molecular drivers and potential therapeutic targets of sarcopenia. Mice are excellent models because of their shorter lifespan, suitability for genetic modification, and physiological similarity to humans. Natural aging is still the most suitable model for studying sarcopenia in rodents as it can resemble the human aging process more closely than genetically modified or senescence-accelerated models (16). However, in natural aging rodent models, sarcopenia is usually categorized by age regardless of sarcopenic status or based only on muscle mass loss. To date, animal studies investigating sarcopenia limit their subject selection to 1 “old” group aged 18–24 months, which is equivalent to humans aged 56–69 years. Given that the life expectancy in the US is approximately 76.1 years old (17), it is crucial to study mice at an equivalent age (24–30 months old in the C57BL/6J strain).

In 2010, sarcopenia in patients was defined as low muscle mass plus low muscle function (muscle strength or physical performance) by the European Working Group on Sarcopenia in Older People (EWGSOP) (18). In 2018, the same group revised this consensus definition for clinical diagnosis (known as EWGSOP2), prioritizing muscle function over muscle mass as the primary parameter for sarcopenia (19). This change was made because muscle strength better predicts adverse outcomes (20–22) and muscle mass is not considered a clinically meaningful outcome, per se, by the Food and Drug Administration and the European Medicines Agency (23). According to this updated sarcopenia definition (19), probable sarcopenia in patients is defined as low muscle strength only (such as hand grip strength); sarcopenia is defined as low muscle strength plus low muscle mass; and severe sarcopenia is defined as the above-mentioned deficits plus low physical performance (such as low walking speed). This definition also supports the need for interventional studies emphasizing the importance of improving muscle function in addition to muscle mass. Despite these recent advances, there is no consensus on the definition of sarcopenia in rodents, which hinders the identification of molecular targets of clinical relevance.

The goals of this study are: (a) to define sarcopenia in C57BL/6J mice of a clinically relevant age range utilizing the new definition of sarcopenia in humans as a model; (b) to understand the relationship between functional loss and muscle mass loss during the progression of sarcopenia; and (c) to elucidate selected molecular mechanisms underlying the progression of sarcopenia. We evaluated the age-related changes in muscle mass and function in young (4–9 months old) and old (23–32 months old) C57BL/6J male mice. We assessed forelimb grip strength, treadmill running time (time to exhaustion), and hindlimb muscle mass (upon termination) to identify sarcopenia in old mice and established normative parameters to define these outcomes. Subsequently, we analyzed muscle physiology and morphological characteristics in these mice and relevant molecular markers to understand their contribution to the progression of sarcopenia. We also performed proteomic analysis in young and old muscles to elucidate the associations between molecular pathways and each measurement of sarcopenia. A group of young (6–7 month-old) and old (27–28 month-old) female mice were also studied for sarcopenia identification and molecular markers to address sex-specific differences in these characteristics.

## Results

### Defining sarcopenia in male and female mice using clinically relevant criteria.

**Figure 1:**
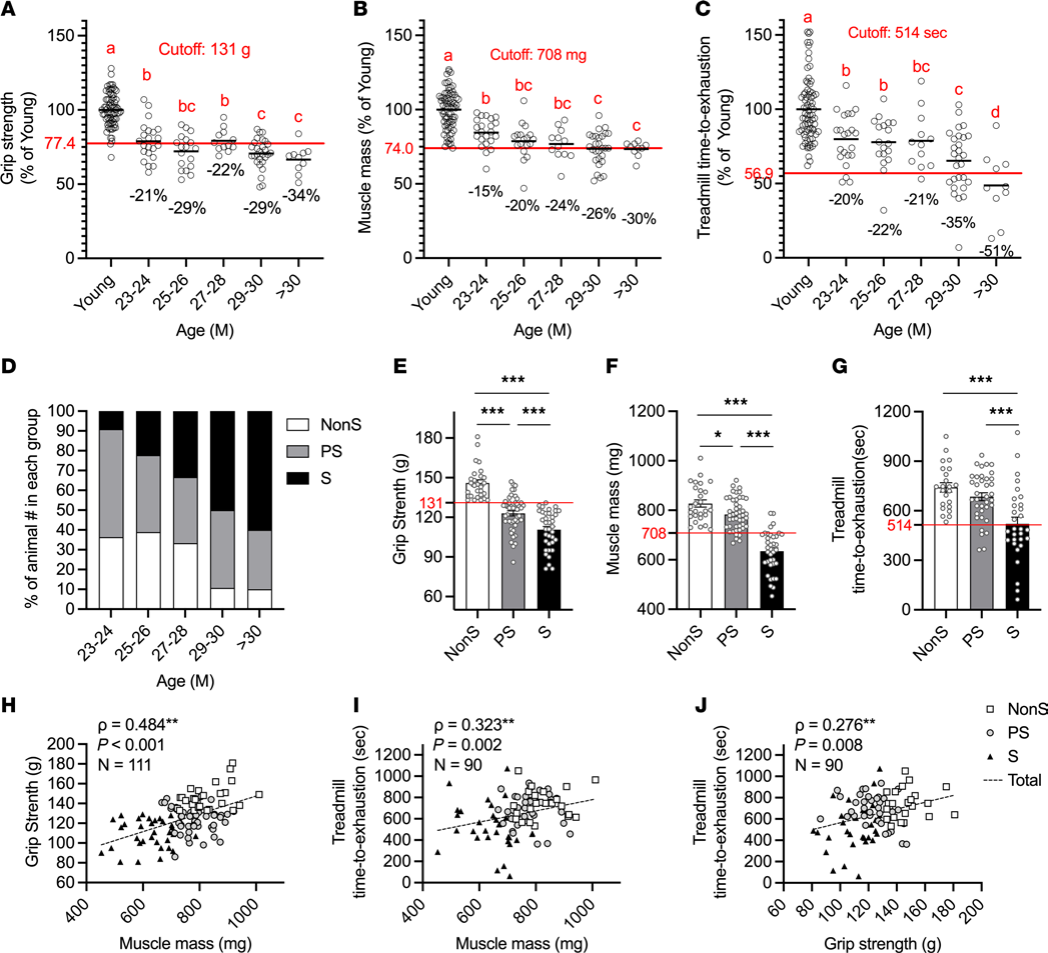
Muscle mass and strength and physical function in young and old C57BL/6J male mice. (**A**) Forelimb grip strength, (**B**) Muscle mass, and (**C**) Treadmill time to exhaustion, by age group, as a percent of the young group’s mean. (**A**–**C**) Red horizontal lines at 2 SDs below the young group’s mean define cutoff points for impairments. 1-way ANOVA with LSD post hoc tests (ANOVA *P* < 0.05) shows significant differences, denoted by different letters (a, b, c, d). (**D**) The percentage of animals in each age group is identified by their sarcopenia status as nonsarcopenic (NonS; 0 deficit), probably sarcopenic (PS, 1 deficit), or sarcopenic (S, 2–3 deficits) based on the numbers of deficits in grip strength, muscle mass, and treadmill running time. (**E**–**G**) Differences in grip strength, muscle mass, and treadmill running time among sarcopenia groups (*n* = 27, 47, 37 for grip strength and muscle mass; *n* = 23, 37, 30 for treadmill). 1-way ANOVA followed by LSD post hoc tests (ANOVA *P* < 0.05) were performed to detect differences among groups (*P < 0.05 and ***P < 0.001 indicate differences in pairwise comparisons). (**H**–**J**) Correlations between grip strength, muscle mass, and treadmill running time in old mice. Correlations were assessed with the Spearman correlation coefficient test (ρ). **P < 0.01.

The EWGSOP2 clinical sarcopenia definition identifies muscle strength as the primary criterion for diagnosing sarcopenia, confirmed by low muscle quality and/or quantity. Severe sarcopenia is diagnosed when low muscle strength, low muscle quantity/quality, and low physical performance are all present (19). To develop a concordant definition in mice, we measured grip strength, total hindlimb muscle mass (milligrams), and treadmill running time (time to exhaustion in seconds) to assess muscle strength, mass, and physical performance, respectively, in young and old male mice. In 23–24 month-old male mice, grip strength and treadmill running time decreased by 21% and 20%, respectively, compared with the young mice, whereas muscle mass decreased by 15%. In mice older than 30 months, the age-related declines were 34% in grip strength, 51% in treadmill running time, and 30% in muscle mass. The decline in treadmill running time from 23 months to over 30 months was more pronounced compared with the other 2 measurements (31% in treadmill versus 13% in grip strength and 15% in muscle mass, Figure 1, A–C). Absolute values of grip strength (g), muscle mass (g), and treadmill running time (s) in male mice at different age brackets are also available in Supplemental Figure 1, A–C; supplemental material available online with this article; https://doi.org/10.1172/JCI172890DS1 Male mice aged 23–26 months showed higher body weight compared with young mice, but differences did not reach significance beyond 26 months (Supplemental Figure 1D). Lean body mass (LBM, which includes skeletal muscle and other organs) increased in old male mice compared with young male mice (Supplemental Figure 1E), and fat mass was 40% higher in male mice aged 23–24 months compared with young male mice but it declined after that. At the age of 29 months or older, fat mass was lower than in young male mice (Supplemental Figure 1F). When grip strength was normalized to body weight (grip strength/body weight, g/g), all old male mice exhibited lower relative grip strength compared with young mice, though no significant age-related difference was observed within the old group (Supplemental Figure 1G). The decrease in grip strength relative to hindlimb muscle mass was more pronounced in male mice aged 23–26 months compared with young male mice. However, in 27–30 month-old male mice, the normalized grip strength was not significantly different from that of the young group (Supplemental Figure 1H). In female mice, at the age of 27–28 months, grip strength, muscle mass, and treadmill running time individually decreased by 13% compared with young mice (Supplemental Figure 2, A–C). In addition, in these old female mice, normalized grip strength (grip strength/BW) decreased by 36% compared with the younger mice (Supplemental Figure 2H), and there was no significant difference after normalizing grip strength to muscle mass (Supplemental Figure 2I). In contrast, body weight, LBM, and fat mass increased by 37%, 19%, and 106%, respectively, in these old female mice compared with their younger peers (Supplemental Figure 2, E–G).

To identify sarcopenia in mice older than 23 months, 3 criteria were used: low muscle strength, muscle mass, and physical performance. Cutoff points were set at 2 SDs below the mean of young mice of the same sex, and a measurement below the cutoff indicated meeting the criterion. This method parallels those used in clinical studies (19, 24). For male mice, the cutoffs were 131 g for muscle strength, 708 mg for muscle mass, and 514 seconds for physical performance (Figure 1, A–C). For female mice, the cutoffs were 126 g, 621 mg, and 576 seconds, respectively (Supplemental Figure 2, A–C). Old mice that did not meet any of the criteria were classified as nonsarcopenic (NonS). Animals that met one criterion were classified as having probable sarcopenia (PS), while animals that met 2 or 3 criteria were classified as having sarcopenia (S). Table 1 shows the percentage of animals exhibiting any of the criteria or a combination of criteria within the same age range. In male mice, the percentage of NonS mice began to decrease at the age of 27–28 months, while the percentage of S mice increased gradually with age (Figure 1D and Table 1). Among old male mice, 25.9% of mice were identified as NonS; 39.2% were identified as PS, with 30.3% exhibiting low grip strength (G), 4.2% with low muscle mass (M), and 4.7% with impaired physical performance (T). Additionally, 34.9% of mice were identified as S, with 14.3% exhibiting both declines in grip strength and muscle mass (GM), 3.6% exhibiting both decreased grip strength and physical performance (GT), and 17% exhibiting declines in all measurements (GMT, Table 1). Significant declines were seen in all 3 measurements among PS or S male mice compared with NonS, except treadmill running time between NonS and PS (Figure 1, E–G). These measurements were positively correlated (Figure 1, H–J). In female mice, the percentages of NonS, PS, and S in old mice were 33.3%, 45.5%, and 21.2%, respectively. Among the female mice with PS, 21.2% had low grip strength (G), 21.2% had low muscle mass (M), and 3% had impaired physical performance (T). Among the female mice with S, 12.1% had both low grip strength and low muscle mass (GM), and 9.1% had all 3 deficits (GMT, Supplemental Figure 2D and Table 1).

### Muscle contractile properties decline as sarcopenia progresses in male mice.

**Figure 2:**
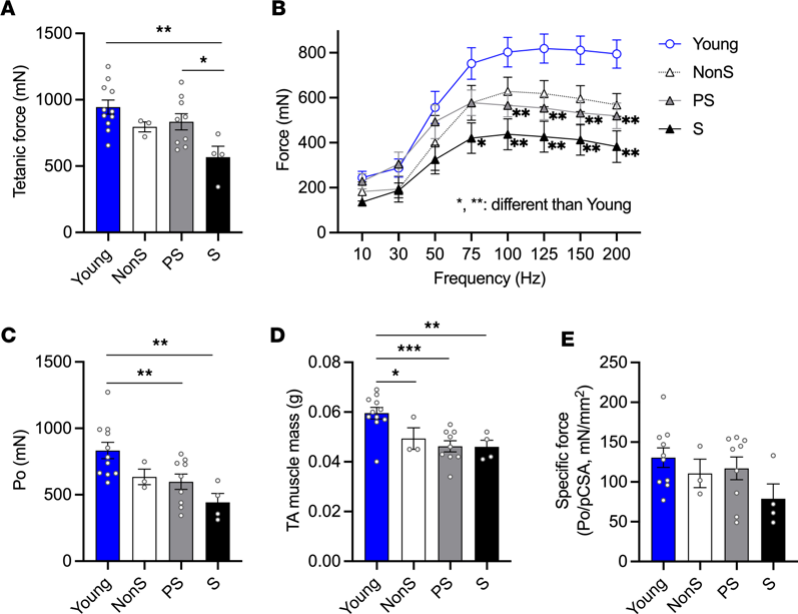
Contractile properties of TA muscles in young and old nonsarcopenic (NonS), probable-sarcopenic (PS), and sarcopenic (S) male mice. (**A**) Tetanic force (mN). (**B**) Contractile force (mN) in response to stimulation at various frequencies (Hz). (**C**) Peak force (Po, mN) was recorded as the highest force production during force-frequency regimen. (**D**) Muscle mass (g). (**E**) Specific force of TA muscle was calculated as peak force divided by physiological cross-sectional area (mN/mm^2^). Data are shown as mean ± SE (*n* = 11, 3, 9, 4). 1-way ANOVA was used to identify differences across groups followed by Dunnett’s (for force/frequency) or LSD post hoc test (ANOVA *P* < 0.05). Tukey HSD post hoc test was conducted when ANOVA was not significant (Specific force, ANOVA *P* = 0.205). *P < 0.05, **P < 0.01, ***P < 0.001 indicate significant differences compared with young in panel B and significant differences between groups for other panels.

To determine if the changes in muscle contractile function worsen with the progression of sarcopenia, we tested in situ muscle contractility in tibialis anterior (TA) muscles in young and old male mice identified as NonS, PS, and S. Mice with S showed significantly lower tetanic force than young mice and old PS mice (Figure 2A). Muscle contractility was conducted by direct stimulation at the TA muscle at gradually increased frequencies every minute. Mice with PS showed declines in force generation starting at 100 Hz, while mice with S showed this decline at 75 Hz (Figure 2B). Consistently, mice with PS and S had a significantly lower peak force compared with the young mice(Figure 2C). An age-related decline was observed in TA muscle mass (Figure 2D), though no difference was found among old mice with different sarcopenia status. Also, mice with S showed numerically lower specific force compared with the young mice, but the difference was not statistically significant (peak force/physiological cross-sectional area, Po/pCSA, Figure 2E).

### Muscle morphological changes in sarcopenia in male mice.

**Figure 3:**
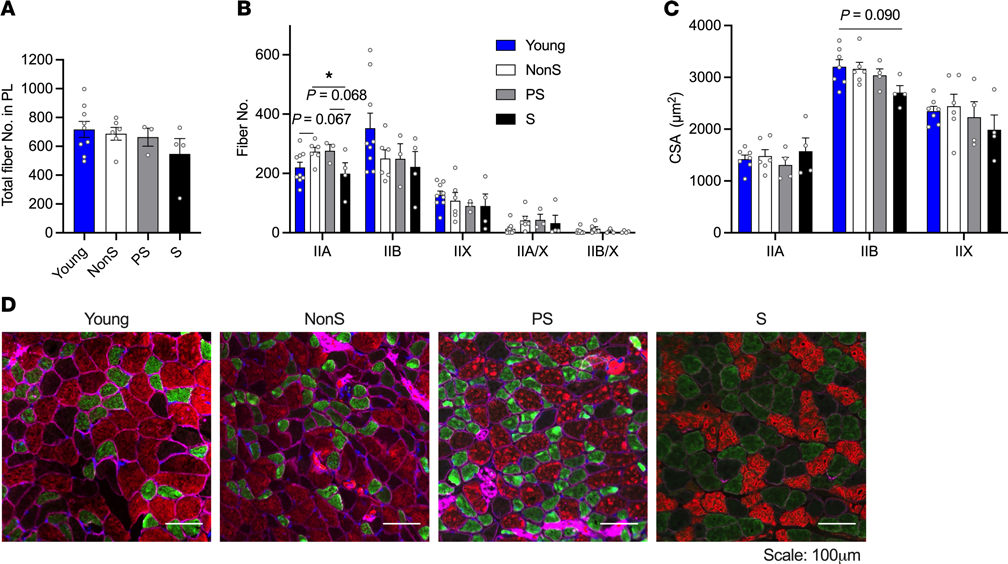
IHC of myosin heavy chain type IIA, IIB, and IIX fibers in PL muscles from young and old NonS, PS, and S male mice (*n*= 9, 6, 3, 4). (**A**) Total fiber number in PL muscles. (**B**) Fiber number in PL muscles by myosin heavy chain (MHC) type (IIA, IIB, IIX, IIA/X, and IIB/X). (**C**) CSA of fibers by MHC type in PL muscles (*n* = 7, 6, 4, 4). (**D**) Representative images of IHC MHC staining for IIA (green), IIB (red), and IIX (no stain, blank) in PL, with membranes stained for dystrophin (magenta). Data are shown as mean ± SE. 1-way ANOVA was used to identify differences across groups followed by LSD post hoc test (IIA fiber number: ANOVA *P* = 0.087, trend difference). Tukey HSD post hoc test was conducted when ANOVA was not significant (other fiber number and CSA, ANOVA *P* > 0.1). *P < 0.05 indicate differences in pairwise comparisons. Trends (0.05 < *P* < 0.1) were marked in the figure with their *P* values. Scale bars: 100 μm.

Fiber number and size CSA in plantaris (PL) muscles were analyzed based on myosin heavy chain (MHC) fiber types (Figure 3, A–D) in male mice. Our results did not show significant differences in total fiber number or fiber numbers based on their MHC fiber types, with the exception of type IIA fibers (Figure 3, A and B. IIA fiber number: ANOVA *P* = 0.087, trend). The number of type IIA fibers tended to be higher in old mice without S compared with young mice (*P* = 0.067); whereas old mice with S showed a lower number of type IIA fibers than nonS or PS old mice (S versus PS: *P* = 0.068, trend). Further, the CSA of IIB fibers tended to be smaller in mice with S compared with young mice (1-way ANOVA: *P* = 0.103, Tukey’s Honestly Significant Difference (Tukey HSD) post hoc test: *P* = 0.09), whereas the CSA of type IIA and IIX fibers remained unchanged regardless of age or sarcopenia status (Figure 3C).

### Impaired mitochondrial function in male sarcopenic mice but unaffected mitochondrial markers in female sarcopenic mice.

**Figure 4:**
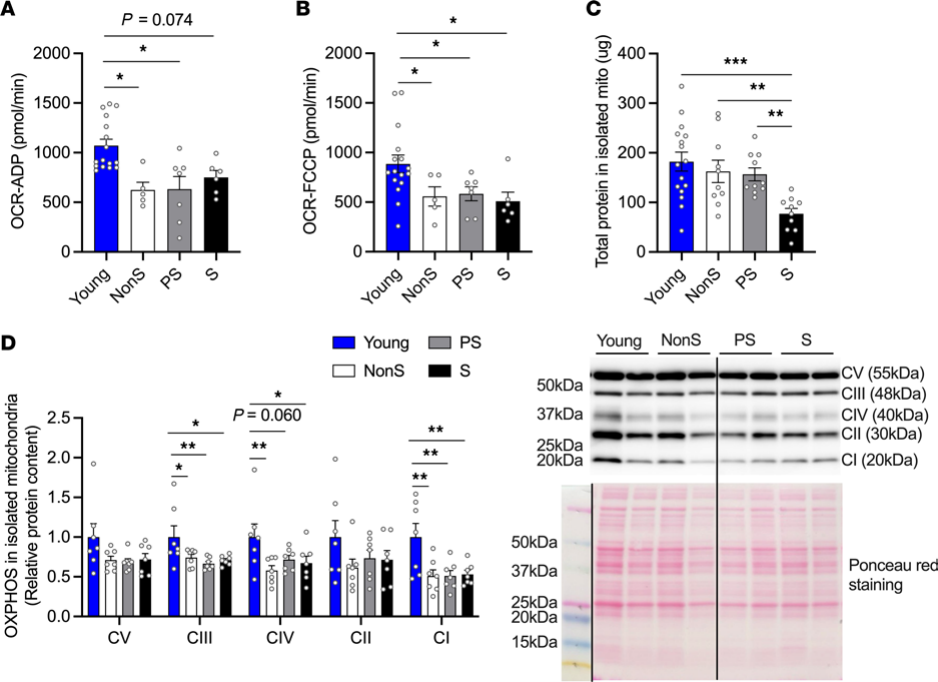
Mitochondrial respiration and OXPHOS complex content in isolated mitochondria from PL muscles of young and old NonS, PS, and S male mice. (**A**) Maximum mitochondrial respiration measured as ADP-stimulated oxygen consumption rate (OCR-ADP, pmol/min, *n* = 16, 5, 7, 6). (**B**) Uncoupled maximum respiration measured as FCCP-stimulated oxygen consumption rate (OCR-FCCP, pmol/min, *n* = 16, 5, 7, 6). (**C**) Total protein in isolated mitochondria (μg) measured by BCA (*n* = 16, 10, 10, 10). (**D**) Protein levels of oxidative phosphorylation (OXPHOS) complexes measured using Western blotting (*n* = 7/group). Western blots were quantified by densitometry and normalized to Ponceau red signal. Representative Western blots and Ponceau red staining are on the right. The line on the blot indicates that lanes are not continuous but from the same blot. Data are shown as mean ± SE. 1-way ANOVA was used to identify differences across groups followed by LSD post hoc test (ANOVA *P* < 0.05). Tukey HSD post hoc test was conducted when ANOVA was not significant (Complex V and II, ANOVA *P* > 0.1). ADP-stimulated oxygen consumption rate was analyzed by Kruskal-Wallis tests as the data was not normally distributed (*P* < 0.05). * *P* < 0.05, ** *P* < 0.01, and *** *P* < 0.001 indicate differences in pairwise comparisons. Trends (0.05 < *P* < 0.1) were marked in the figure with their *P* values.

**Figure 5:**
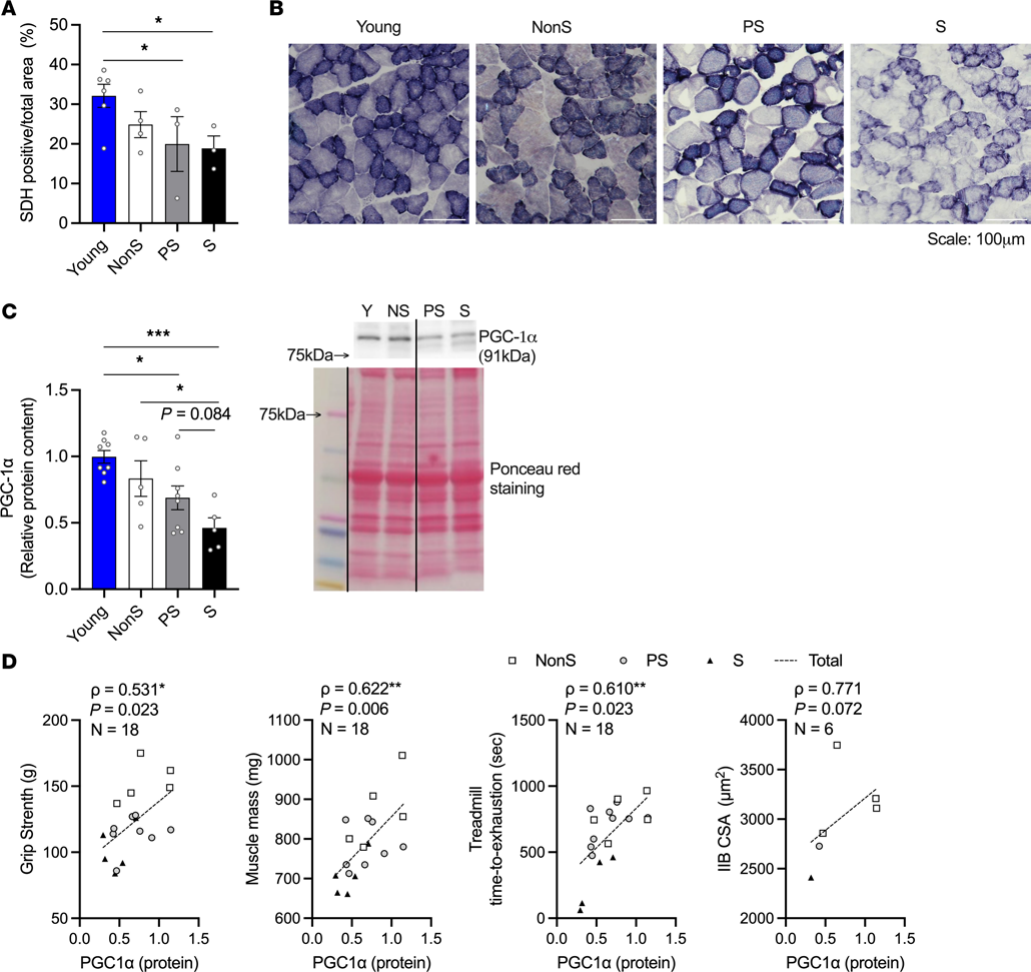
Oxidative capacity and mitochondria biogenesis markers in PL muscles of young and old NonS, PS, and S male mice. (**A**) Percentage of SDH-positive area in PL muscles (*n* = 6, 4, 3, 3). (**B**) Representative images of SDH-stained PL muscles. Scale bars: 100 μm. (**C**) Relative protein content of PGC-1α in GAS/PL muscles (*n* = 8, 5, 8, 5), measured by Western blotting, quantified by densitometry, and normalized to Ponceau red signal. Representative Western blots and Ponceau red staining are shown on the right. The line on the blot indicates that lanes are not continuous but from the same blot. Data are shown as mean ± SE. (**A** and **C**) 1-way ANOVA was used to identify differences across groups followed by LSD post hoc test (ANOVA *P* < 0.05 for PGC-1α; ANOVA *P* = 0.095 for SDH, trend difference). *P < 0.05, **P < 0.01, and ***P < 0.001 indicate differences in pairwise comparisons. Trends (0.05 < *P* < 0.1) are marked in the figure with the *P* value. (**D**) Correlations between PGC1-α protein content and grip strength, muscle mass, treadmill running time, or MHC IIB fiber cross-sectional area. Correlations were assessed with the Spearman correlation coefficient test (ρ). *P < 0.05, **P < 0.01.

Mitochondrial function is central to maintaining muscle quality during aging (25, 26). We assessed mitochondrial respiration by examining isolated mitochondria in the PL muscles. Maximum mitochondrial respiration, including ADP-stimulated (oxygen consumption rate, OCR-ADP) and uncoupled maximum respiration (OCR-FCCP), was reduced in old mice, regardless of their sarcopenic status (Figure 4, A and B). The total protein content of isolated mitochondria from PL muscles was significantly lower in the S group compared with the other groups (Figure 4C). Compared with the young group, the content of oxidative phosphorylation (OXPHOS) complexes III and I showed a significant decrease in old mice, regardless of their sarcopenic status. The content of complex IV followed the same pattern in which old mice showed lower content than young mice, with the difference between the young and PS groups only approaching significance (*P* = 0.06). Complexes V and II contents showed similar patterns to other complexes, but there were no significant differences. There was no difference in any of the OXPHOS complexes among the old mice regardless of their sarcopenic status (Figure 4D). To further identify oxidative capacity changes during sarcopenia, we analyzed the succinate dehydrogenase (SDH) positive area in PL muscles, a marker of complex II–regulated oxidative capacity (Figure 5, A and B). With a trend in 1-way ANOVA (*P* = 0.095), the SDH-positive area was lower in PS and S mice compared with the young mice, whereas mice with NonS were not different from any of the groups (Figure 5, A and B). At the whole muscle level, the protein content of peroxisome proliferator–activated receptor (PPAR) γ coactivator 1α (PGC-1α), a marker of mitochondrial biogenesis, decreased in S mice compared with NonS and young mice, and an age-related decrease in this protein was also observed in PS mice (Figure 5C). We found positive correlations between PGC-1α levels and grip, treadmill, muscle mass, and IIB CSA (trend only) in old mice (Figure 5D). In female mice, the protein levels of PGC-1α in gastrocnemius (GAS) muscles and total protein levels of isolated mitochondria from PL muscles showed no differences across different groups (Supplemental Figure 3, A and B). Similar results were observed for OXPHOS complexes levels in isolated mitochondria (Supplemental Figure 3C).

### Autophagy and AMPK signaling in sarcopenia in male and female mice.

**Figure 6:**
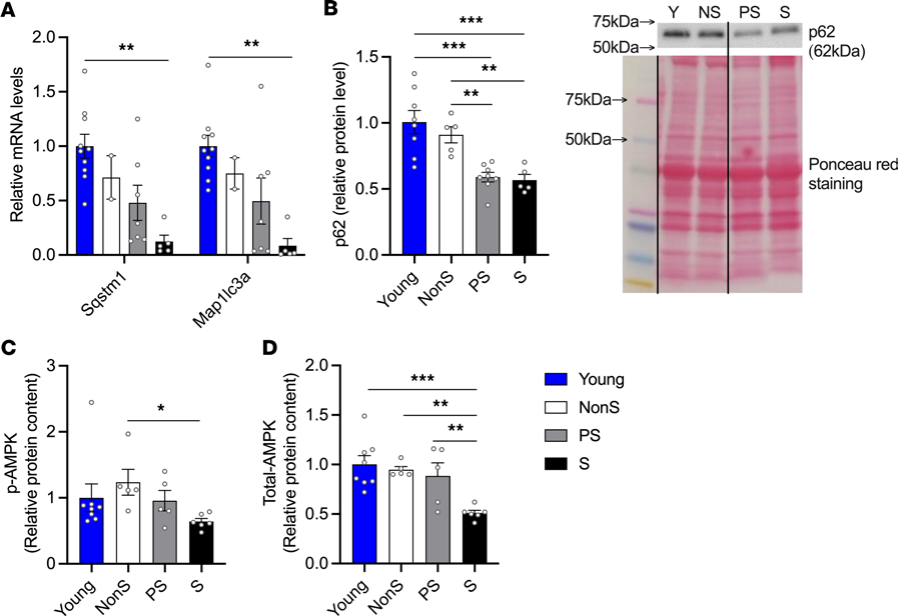
Molecular markers for autophagy and AMPK signaling in skeletal muscles in young and old NonS, PS, and S male mice. (**A**) Gene expression of autophagy markers *Sqstm1* (p62) and *Map1lc3a* (LC3) (*n* = 10, 3, 7, 5) as measured by RT-qPCR. *Hprt* was used as a reference gene and data are expressed as relative mRNA fold-change from the young group. (**B**) Relative protein level of the autophagy marker p62 (*n* = 8, 5, 8, 5) in GAS/PL muscles as measured by Western blotting, quantified by densitometry, and normalized to Ponceau red signal. Representative Western blots and Ponceau red staining are shown on the right. The line on the blot indicates that lanes are not continuous but from the same blot. (**C**) Relative protein content of p-AMPKα (Thr172) and (**D**) total AMPK (*n* = 8, 5, 5, 6) as measured in Quad muscles by MSD electrochemiluminescence immunoassay. Data are shown as mean ± SE. 1-way ANOVA was used to identify differences across groups followed by LSD post hoc test (ANOVA *P* < 0.05). Gene expression of autophagy markers and p-AMPKα protein levels were analyzed by Kruskal-Wallis tests as the data was not normally distributed (*P* < 0.05). *P < 0.05, **P< 0.01, and ***P < 0.001 indicate differences in pairwise comparisons.

Autophagy serves as a quality control mechanism that breaks down damaged organelles, including mitochondria (mitophagy), as well as the endoplasmic reticulum (ER) and ribosomes (25). In male mice, we found decreases in the mRNA levels of sequestosome-1 (*Sqstm1*/p62) and microtubule-associated proteins 1A/1B light chain 3A (*Map1lc3a*/LC3) in muscles from S mice compared with young mice (Figure 6A). The protein level of p62 was lower in PS and S mice than in young and NonS mice (Figure 6B). We further investigated 5′ AMP-activated protein kinase (AMPK) and phosphorylated-AMPK (p-AMPK) levels in skeletal muscles in male mice, which play a role in regulating autophagy (27). A significant decrease was observed in p-AMPK (Th172) in S mice compared with the NonS group. Total AMPK levels were also lower in S mice than in all the other groups (Figure 6, C and D). In female mice, no significant differences were detected in protein levels of p62 or AMPK (both phosphorylated and total) among all groups (Supplemental Figure 3, D–F).

### Lack of increase in atrogenes with sarcopenia in male or female mice.

**Figure 7:**
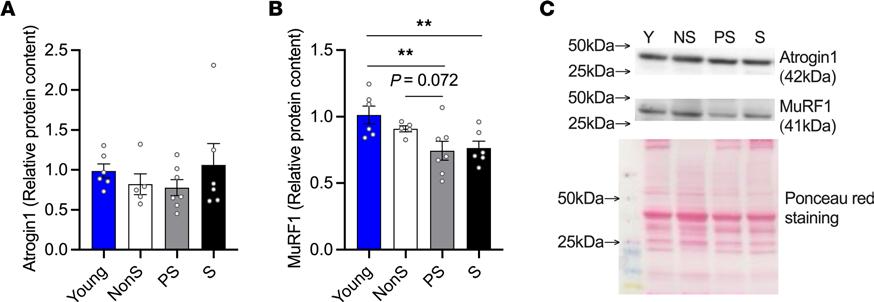
Relative protein content of atrogenes. (**A**) Atrogin1 and (**B**) MuRF1 in GAS/PL muscles of young and old NonS, PS, and S male mice *n* = 6566, as measured by Western blotting, quantified by densitometry, and normalized to Ponceau red signal. (**C**) Representative Western blots and Ponceau red staining. Data are shown as mean ± SE. 1-way ANOVA was used to identify differences across group means followed by LSD post hoc test (ANOVA *P* < 0.05). *P < 0.05 and **P < 0.01 indicate differences in pairwise comparisons. A trend (0.05 < *P* < 0.1) was marked in the figure with the *P* value.

The E3 ligases Atrogin1 and Muscle Ring Finger Protein–1 (MuRF1), also known as atrogenes, are responsible for ubiquitin-proteasome–regulated protein degradation during muscle wasting. To understand its role in the progression of sarcopenia, we tested their protein levels in skeletal muscles in male mice (Figure 7, A–C). There was no significant difference in Atrogin1 protein levels between groups (Figure 7A). MuRF1 levels were lower in the muscles of PS and S mice than in young mice, and a trend of decrease in MuRF1 was also observed in PS mice compared with the NonS group (*P* = 0.072; Figure 7B). In female mice, no significant differences were detected in protein levels of atrogenes among all the groups (Supplemental Figure 3, G and H).

### KEGG pathways are correlated with muscle mass and function differently in male mice.

To elucidate the molecular mechanisms underlying the progression of sarcopenia, we conducted proteomic analyses on GAS muscles of young (6–7 months old) and old (27–29 months old) male C57BL/6J mice. As a result, we identified 3,110 protein groups mapped from 24,483 detectable peptides within the GAS muscles in young and old mice. Linear regression analyses were then carried out separately for young and old mice to explore potential correlations between these proteins and any of the sarcopenia measures, including grip strength, muscle mass, or treadmill running time. No significant correlations were observed between the identified proteins and sarcopenia measurements at the individual protein level (data not shown). We then investigated whether specific molecular pathways were associated with the progression of sarcopenia. We performed a competitive gene set test (details in the Method section) and a main effect analysis to identify Kyoto Encyclopedia of Genes and Genomes (KEGG) pathways that showed similar patterns in young and old mice in terms of correlations between protein abundance and sarcopenia measurements (“+” and “–” represent positive and negative correlations, respectively. FDR < 0.05). We found that proteins in 11 KEGG pathways were positively correlated, and 2 pathways were negatively correlated with muscle mass. These positively correlated pathways include OXPHOS, thermogenesis, citrate cycle, and metabolic processes for fatty acids, carbon, and amino acids. Proteins in complement and coagulation cascades were negatively correlated with muscle mass but positively correlated with treadmill running time (Table 2). No significant main effect was observed in correlations between any KEGG pathways and grip strength. The top 5 proteins contributing to these correlations within each KEGG pathway are shown in Table 2.

## Discussion

Interventions for sarcopenia, the progressive loss of muscle mass and function with aging, are still lacking, and the molecular underpinnings of muscle mass and function loss in this setting are incompletely understood. Despite the importance of animal studies in elucidating the mechanisms leading to sarcopenia, a clear definition of sarcopenia in preclinical studies has not been established. Further, mouse models that reflect the progression of sarcopenia in equivalent human age are still lacking. To our knowledge, this is the first study to identify sarcopenia using a clinically relevant definition in male C57BL/6J mice aged 23–32 months and female C57BL/6J mice aged 27–28 months, which are equivalent to humans aged 66 to 86 years old, and 80 years old, respectively (28). In male mice, with this definition, there are distinct trajectories for changes in muscle mass, strength, and functional performance that are associated with molecular alterations in mitochondrial function, autophagy, and AMPK signaling, but not in atrogenes. With proteomic analysis in male muscles, we confirmed a positive correlation between muscle mass and protein abundance in mitochondrial metabolism pathways, and pathways related to fibrosis and immune responses were associated with modifications in treadmill running time. In contrast, in female mice, the progression of sarcopenia is less pronounced, and their body fat is higher compared with male mice. Unlike male mice, they exhibit a milder sarcopenia phenotype without significant changes in mitochondrial biogenesis, OXPHOS complex content, AMPK signaling, mitophagy, or atrogenes.

With aging, the decrease in muscle function is greater than in muscle mass (3, 29). After the age of 50, the decline in leg lean body mass is about 1%–2% per year in humans (30–33) while strength loss is 1.5%–5% per year (30, 31). In male mice, the age-related decrease in muscle mass was not as prominent as the declines in muscle strength (grip) and physical performance (treadmill running time) at early stages of aging (23–24 months), and physical performance declines more dramatically than muscle mass and strength in the late stages of aging (over 30 months). A similar finding has also been reported in a longitudinal study of frailty status in C57BL/6J mice, where the age-related decline in treadmill running time was more prominent than the decrease in grip strength in mice aged 23–32 months (34). When grip strength was normalized to body weight, all old groups showed decreased grip strength, likely due to the 23–26 month group having higher body weight than older groups. Normalizing grip strength to hindlimb muscle mass, which more accurately represents muscle mass (35), still showed a decrease in grip strength in 23–26 month groups, but to a lesser extent. Further, higher muscle mass was moderately correlated with higher muscle strength in old mice (ρ = 0.48), suggesting muscle mass is not the sole contributor to the decline in muscle strength, particularly in mice between the ages of 23 and 26 months. In mice older than 27 months, the decrease in muscle mass may be a more important factor leading to strength decline. Female mice aged 27–28 months exhibited less age-related deteriorations in grip strength, muscle mass, and physical performance and had higher fat mass compared with same-age males. These findings align with previous clinical and preclinical studies regarding age-related changes in muscle loss and body composition (2, 36, 37). Despite inconsistent findings on the association between adiposity and frailty and/or mortality across various studies and cohorts in female mice (36, 38), we did not observe a higher prevalence of sarcopenia in older females with higher body fat compared with males of the same age. Future studies should investigate the association between body fat and sarcopenia stages in mice.

We utilized mice aged between 4 and 9 months as the young standard because this age range is equivalent to a human age range of approximately 23 to 36 years old (28). In the definition of sarcopenia in humans, healthy young adults aged 18 to 40 years old have been commonly used as a reference group in many studies. For instance, the standard for Skeletal Muscle Index (SMI), calculated as the skeletal muscle mass relative to total body mass, has been established in individuals aged 18 to 39 years (39). Similarly, the definition of low grip strength was derived relative to the peak mean values observed in humans, which occur between 29 and 39 years old in men and 26 and 42 years old in women (24). We also performed a sensitivity analysis restricting the age range to 4–6 months old, but this did not alter the overall results (data not shown). In our current study, we defined probable sarcopenia as exhibiting 1 deficit and sarcopenia as showing deficits in at least 2 out of the 3 sarcopenic criteria: grip strength, muscle mass, and physical performance (treadmill running time). Using our proposed cutoff points (at least 2 SDs below the mean in young mice), the prevalence of sarcopenia was 9%–22% in male mice aged 23–26 months and 21.2% in female mice aged 27–28 months. This is similar to the prevalence of sarcopenia in humans of an equivalent age (over 60 years old), which ranges from 10%–27% (40), and confirms the translational importance of this definition. In clinical studies, hand grip strength is currently considered the primary parameter in diagnosing sarcopenia because it is a strong predictor for prolonged hospitalization, increased functional limitations, poor quality of life, and increased mortality (21, 22, 41–44). Interestingly, we found that the majority of male mice defined as probably sarcopenic had low grip strength rather than low muscle mass or low physical performance. In addition, most of the male mice with sarcopenia showed a combination of all 3 criteria, followed by low grip strength plus muscle mass, and low grip strength plus physical performance, whereas there was no case of only low muscle mass plus physical performance. These findings suggest that low grip strength presents earlier than the other 2 criteria in sarcopenia, further supporting the concept that low muscle strength can be considered an early and central parameter for sarcopenia diagnosis in male mice. Our data also shows that muscle strength is more closely related to muscle mass than physical performance in male mice.

Declines in muscle quantity (muscle size) and quality (muscle strength and/or power per unit of muscle mass) are responsible for age-related impairments in muscle contractility (45, 46). Clinically, the decline in muscle quality is associated with age-related changes in glucose and protein metabolism, modifications to muscle structure, oxidative stress–induced damage, adipose infiltration, decreased capillary density, and alterations in both contractility and fatigability (47). In preclinical sarcopenia studies, a commonly used method to detect muscle quality is relative force production (47, 48), including specific force (maximum force normalized to muscle cross-sectional area). Studies in sarcopenia using mouse models have reported both decreased and maintained specific force. Graber et al. and Qaisar et al. reported decreased specific force in the extensor digitorum longus (EDL) muscles of 25–28 month-old mice compared with 6–10 month-old mice, with Graber’s group also noting similar changes in the soleus muscles (49, 50). In contrast, Ham et al. found that 30-month-old mice maintained specific force in soleus and EDL muscles compared with 10-month-old mice (51). Similarly, Weber et al. observed that specific force in plantar flexor muscle groups was maintained in 26-month-old mice compared with 11-month-old mice (52). The variation in age-related changes in specific force between studies may be influenced by differences in the age or sarcopenic status of the mouse cohorts studied. Although we found a decrease in muscle mass in all aged muscles regardless of their sarcopenic status at 27–28 months in male mice, specific force in sarcopenic mice was numerically lower than in the young group. This suggests that both muscle quantity and, to a lesser degree, quality loss may contribute to the impairment in muscle contractility with age. More studies will be needed to confirm this hypothesis. Our in vivo data show a moderate correlation between muscle mass and strength in old mice, suggesting that the impairment in muscle quality (specific force) is a crucial contributor to the age-related decline in muscle strength. Some of the mechanisms thought to contribute to a reduced specific force include an imbalance in calcium homeostasis, disrupted excitation-contraction (EC) coupling, and actin-myosin interaction (45, 46, 53). More studies will be needed to establish the contribution of these mechanisms to mice with sarcopenia.

Consistent with the literature, we found that fast-twitch fibers were more vulnerable to age-induced changes than slow-twitch fibers, and a fast-to-slow fiber-type transformation has been observed during the aging process (11, 12). This fiber-type shifting also contributes to impaired contractile function, especially muscle power and velocity (12, 54). This may explain our finding of a high prevalence of low grip strength during sarcopenia, as it is likely due to age-related preferential atrophy and loss of type IIB fibers. With the progression of sarcopenia, we found decreased oxidative capacity and PGC-1α levels despite maintaining slow-twitch fiber size. Although PGC-1α is known for maintaining oxidative fibers (55) and has a negative effect on fast-twitch fibers in adult mice (56, 57), diminishing PGC-1α expression in old mice also induces IIX and IIB fiber atrophy (58). Consistently, in our old male mice, type IIB fiber size tended to be associated with PGC-1α levels; it is likely that maintaining slow-twitch fiber size alone is insufficient to compensate for age-related loss in oxidative capacity in sarcopenic mice.

Age-related impairment in mitochondrial function has been proposed as a contributor to the decline in muscle function (15, 26, 59). With aging, we observed a decrease in mitochondrial respiration and OXPHOS content normalized to mitochondrial protein, independent of sarcopenic status in male mice. However, at the whole-tissue level, mitochondrial protein content and oxidative capacity decrease with the progression of sarcopenia. PGC-1α regulates mitochondrial biogenesis and oxidative metabolism, which is crucial for providing energy during muscle contraction and protects muscles from proteolysis, oxidative damage, inflammation, autophagy, and apoptosis (60). Low PGC-1α levels are seen in older adults (61) and aged rodents (62, 63). PGC-1α muscle-specific knockout mice exhibit reduced endurance capacity (56), whereas PGC-1α overexpression improves muscle oxidative capacity and fatigability in 24-month-old mice (64) as well as muscle endurance (58). We showed decreased PGC-1α levels in sarcopenic mice and a positive correlation between its content and all 3 measurements of sarcopenia, suggesting that PGC-1α is important for the progression of sarcopenia. In addition, our proteomic analysis showed that proteins involved in mitochondrial metabolism pathways have a consistent positive correlation with muscle mass across all age groups, including OXPHOS, the citrate cycle (TCA cycle), 2-oxocarboxylic acid metabolism, and thermogenesis. This highlights the central role of mitochondrial metabolism in regulating muscle mass in both young and old male mice, aligning with previous studies using different omics technologies and showing age-related downregulation of mitochondrial components and metabolism in skeletal muscles from mice (59, 65, 66). Our results support the hypothesis that, while aging itself decreases mitochondrial function in skeletal muscle, sarcopenia develops when mitochondrial protein content and PGC-1α decline, impairing oxidative capacity at the whole tissue level. This association between healthy mitochondrial metabolism and muscle mass is particularly evident in male mice. In female mice, the declines in grip, muscle mass, and endurance were comparatively milder, and we did not observe age- or sarcopenia-related decreases in PGC-1α and OXPHOS complexes as in male mice. In young mice, females normally have higher mitochondrial content than males (67), which is related to a higher number of oxidative fibers (68) and a predominant role of fat oxidation to produce ATP (69); with aging, the declines in mitochondrial content and OXPHOS complexes has been mainly observed in male but not females mice (65, 67). Thus, these sex-specific differences in mitochondrial content and age-related mitochondrial dysfunction could partially explain our observation of unchanged mitochondrial content or OXPHOS complexes in female mice with a less severe form of sarcopenia. Nevertheless, it has been reported that the quality of mitochondria in female mice deteriorates with age, independent of mitochondrial content, leading to decreases in ATP production and increases in H_2_O_2_ production (65). Future studies focusing on mitochondrial function and the quality of mitochondria will be important to elucidate their correlation with the progression of sarcopenia in female mice. It is also possible that sarcopenia is influenced by other aspects of mitochondria, such as mitochondria integrity, fusion and fission, and reactive oxidative species production (15, 65). More studies will be required to test these hypotheses.

Although excessive autophagy activation has been associated with muscle atrophy, such as cachexia (70, 71), conversely, autophagy also has a protective role in removing damaged DNA, proteins, and mitochondria (mitophagy). In 26-month-old mice and sedentary men, a decline in autophagy markers ATG7 and LC3II to I ratio has been observed, indicating a decrease in autophagy activity (72). We found that old male mice with sarcopenia had particularly low autophagy marker levels compared with young and/or old mice without sarcopenia. AMPK activation can restore mitochondrial function by activating autophagy via ULK1 to remove damaged mitochondria and reduce mitochondrial oxidative stress (27, 73). AMPK-autophagy signaling can be activated by caloric restriction (74) and exercise (72, 75), and both interventions have shown promise in improving age-related decline in muscle strength. In our model, we observed decreased phosphorylated AMPK (Thr172) and total AMPK levels in sarcopenic male mice compared with old male mice without sarcopenia, suggesting an important role of AMPK-autophagy signaling in maintaining muscle mass and function during aging. In contrast, in female mice, the mitophagy marker p62 level was similar in older mice during the progression of sarcopenia compared with young mice. Elevated autophagy (Atg7) and mitophagy markers (Parkin) with aging in skeletal muscle have been reported in female mice at 22–24 months old, in which this elevation is also more prominent than in their male counterparts (67). It is possible that maintained mitophagy in female mice contributes to the preservation of grip strength, muscle mass, and treadmill running time compared with same-aged male mice. Further studies will be necessary to validate this hypothesis.

Atrogin1 and MuRF1 are 2 ubiquitin E3 ligases targeting proteins tagged with polyubiquitin and bringing them to the 26S proteasome for degradation. In sarcopenia, increased, decreased, or unchanged mRNA levels of MuRF1 and Atrogin1 with aging have been reported in studies using rats or human skeletal muscles (reviewed in ref. 76). In mouse models, there is no change in Atrogin1 or MuRF1 levels in the gastrocnemius complex from 24 month-old WT mice compared with 6 month-old mice (77). Inhibition of MuRF1 improved muscle mass in old mice but failed to protect against age-related decline in muscle contractile function, whereas atrogin1 knockout mice exhibited shortened lifespans (77). In male mice, we did not observe any elevation in MuRF1 and Atrogin1 levels with aging or the progression of sarcopenia, suggesting the activation of proteolysis is not a major player in sarcopenia. On the contrary, we observed a decrease in MuRF1 levels with the progression of sarcopenia but no change in Atrogin1, suggesting these 2 E3 ligases may play different roles in aging in male mice. MuRF1 facilitates the degradation of muscle structural and contractile proteins, such as troponin1, myosin heavy and light chains, and myosin-binding protein C (78), as well as neuromuscular junction (76, 78). In contrast, Atrogin1 negatively regulates protein synthesis by inhibiting signaling pathways involving mTORC1/eIF3f/S6K1 signaling and myogenesis (79). While both Atrogin1 and MuRF1 share common transcription factors, such as the class O-type forkhead transcription factors (FOXOs), there are also specific transcription factors for Atrogin1 including C/EBP-β, TGF-β, and Smad3 (80, 81). More studies are needed to investigate the involvement of different E3 ligases and these mediators in sarcopenia. Young female mice tend to favor autophagy-regulated catabolism over ubiquitin proteasome-regulated proteolysis in basal conditions compared with their male counterparts, and this sex difference is believed to be modulated by estrogen signaling (82, 83). We observed no alterations in Atrogin1 and MuRF1 levels in female muscles with aging, nor during the development of sarcopenia. These results suggest that sex-specific differences in protein catabolism persist with the progression of sarcopenia in old mice. Taken together, our data suggest that the mechanisms underlying the development of sarcopenia in male and female mice are different and that preservation of mitochondrial function along with mitophagy may mediate the preservation of muscle mass and function during aging in female mice.

Our proteomic analysis in male muscles also showed the complement and coagulation cascades pathway exhibits a negative correlation with muscle mass but a positive correlation with treadmill running time. This discrepancy can be attributed to differences in the top-ranked proteins associated with each correlation. For instance, proteins such as C1QA, C1QC, CO8B, FIBA, and FIBB, involved in complement and fibrogenesis, are the top-ranked proteins in the correlation with muscle mass. These proteins activate Wnt signaling and promote fibrosis (84, 85), which is a process known to be elevated within aging muscles. Conversely, serpina proteins (A1AT1, A1AT2, A1AT3, and A1AT4) dominate the correlation with treadmill running time, known for their antiprotease and antiinflammatory effects (86). These findings suggest that age-related increases in fibrosis may contribute to decreased muscle mass, whereas antiprotease and antiinflammatory activities are associated with endurance.

This study has several limitations. First, the study mainly focused on male mice, we only studied a smaller age range for female mice (27–28 month-old) with limited molecular markers. However, we have addressed several sex differences in the progression of sarcopenia, body composition, and molecular changes in mitochondrial markers and protein catabolic markers within this age range. To better understand sex differences in the development and progression of sarcopenia, future studies should include a wider age range of female mice. These studies should also evaluate morphological changes and contractility function, as differences between sexes have been observed previously in these characteristics (87, 88). Additionally, employing proteomics or RNA-Seq techniques in female mice may offer a more comprehensive understanding of the underlying molecular mechanisms. Secondly, our study was cross sectional, and it is possible that survivor effects could have influenced the results in mice at older ages. Third, our sample size was relatively small, and we only provided a snapshot of the molecular changes occurring in these mice. Moreover, in the current study, we only used hindlimb muscle mass as the measurement of low muscle mass, which is the most commonly used muscle in rodent studies (16, 59) but it does not represent total muscle mass including trunk muscles. However, we still consider this method to have translational relevance since lower limb muscle dysfunction is a key contributor to impaired physical function and poor quality of life in humans (9, 89). Also, mouse and human GAS muscles undergo similar age-related changes in mitochondrial function, RNA processing, and autophagy (59). Hence, studying hindlimb muscles in the present study will contribute crucial data to other studies using mice as a model of sarcopenia. Lastly, another biological syndrome highly associated with sarcopenia is frailty, characterized by increased vulnerability to adverse health outcomes, such as falls, hospitalization, disability, and mortality, when exposed to stressors (90). With aging, sarcopenia and frailty emerge together and form a vicious cycle (91). Clinically, the prevalence of sarcopenia is usually higher than that of frailty (33, 92), and sarcopenia is considered a precursor of frailty (93). A classic definition of the frailty phenotype established by Fried and colleagues also includes hallmarks of sarcopenia, such as weakness, slow walking speed, and exhaustion, in addition to weight loss and low physical activity (94). In animal studies, the relationship between sarcopenia and frailty is less clear. A Frailty Index was originally developed for mice based on Fried frailty phenotype criteria (95) and has proven to predict mortality rates in old C57BL/6 mice (34, 38). As in humans, the definition of sarcopenia in mice proposed here resembles the Frailty Index, as both were derived from established clinical definitions and both used treadmill running and grip tests as assessments for physical performance. However, there are also differences between the 2 sets of assessments: The Frailty Index utilizes cutoff points derived from the mean of an older cohort, while the sarcopenia definition in current study references young mice with the same background as a standard. Additionally, the Frailty Index includes voluntary wheel running, rotarod tests, and body weight, whereas the sarcopenia definition requires a terminal criterion of hindlimb muscle mass. Future studies should include both assessment tools to have a better understanding of the relationship between sarcopenia and frailty in animal studies.

In conclusion, our study proposes a definition of sarcopenia in mice that is clinically relevant and has substantial translational value. Our findings shed light on the distinct trajectories of muscle mass and function loss during aging and demonstrate the differential effects of aging and sarcopenia on molecular markers of mitochondrial function, autophagy, and AMPK signaling. Overall, our study provides important insights into the pathophysiology of sarcopenia and sets the basis for further research in this area.

## Methods

### Sex as a biological variable.

Our study used both male (4–9 and 23–32 month-old) and female (6–7 and 27–28 month-old) C57BL/6J mice to evaluate sarcopenia and subsequent molecular analysis. This approach allowed us to evaluate sex-specific differences in the progression of sarcopenia and gain deeper insights into the distinct molecular signaling pathways involved.

### Animals.

The original C57BL/6J line was from Jackson Laboratory. All C57BL/6J mice in the current study were born and raised in the vivarium at VA Puget Sound Health Care System. Mice were categorized by sarcopenic status based on functional tests and muscle mass as described in the Results and Supplemental Methods. Mice were individually housed and maintained on a 12/12 light/dark cycle (lights on at 6 a.m.).

Other methods are described in Supplemental Methods.

### Statistics.

The statistical analysis was adapted from the methods described by Morgan (96). In brief, we conducted Shapiro-Wilk normality tests on all parameters within each sarcopenic group. If the parameters demonstrated normal distribution within each group, we used 1-way ANOVA for analysis. If the ANOVA results were statistically significant (*P* < 0.05) or showed a trend toward significance (*P* < 0.1), we proceeded with Fisher’s Least Significant Difference (LSD) test unless otherwise stated. If the *P* value from the ANOVA test was greater than 0.1, we conducted a Tukey HSD post hoc test and reported that *P* value. For parameters that did not exhibit normal distribution, we conducted a reanalysis using nonparametric tests (Kruskal-Wallis). For all the above-mentioned tests, we consider results with a *P* < 0.05 to indicate significant differences. Additionally, results showing a *P* < 0.1 are interpreted as indicating a trend toward significance. Spearman correlations were used to determine associations (*P* < 0.05). 2-tailed independent *t* tests were used to compare age differences in female mice (*P* < 0.05). Values are presented in mean ± SE. All statistical testing except for proteomic analysis was performed using IBM SPSS version 18 software. Statistical analysis for abundance proteomics is described in Supplemental Methods. GraphPad Prism software was used for data visualization.

### Study approval.

All experiments were conducted with the approval of the Institutional Animal Care and Use Committee at VA Puget Sound Health Care System (Protocol no. 0950) and in compliance with the NIH Guidelines for Use and Care of Laboratory Animals.

### Data availability.

Values for all data points associated with the manuscript and supplemental results are provided in the Supporting Data Values document. Data availability for abundance proteomics is described in Supplemental Methods. Additional information and detailed methods in this paper will be available upon direct request from the authors.

## Author contributions

HLK and JMG conducted the study design. HLK, KK, LC, GEM, JWM, TKB, and JMG performed data analysis, interpretation, and manuscript preparation. HLK, KK, BA, LC, and GEM performed biochemical experiments and data collection. A Christiani and LS conducted animal behavioral tests. TL, BI, SJ, AR, A Chen, and ED assisted in terminal surgeries and performed immunohistochemical quantification. MJM provided guidance and resources for proteomic analysis. All authors reviewed and approved the final version of the manuscript.

## Supplementary Material

Supplemental data

Unedited blot and gel images

Supporting data values

## Figures and Tables

**Table 1:**
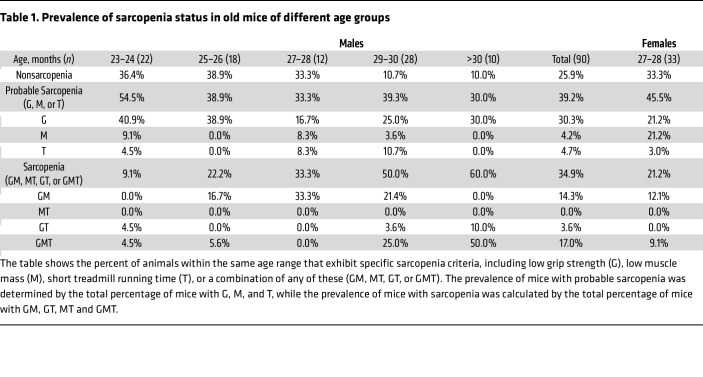
Prevalence of sarcopenia status in old mice of different age groups

**Table 2:**
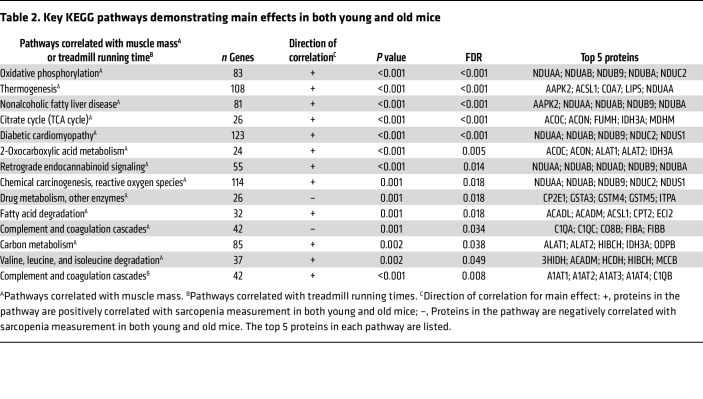
Key KEGG pathways demonstrating main effects in both young and old mice

## Version 1 08/15/2024

Electronic publication
