# Supplementary material for: Mouse sarcopenia model reveals sex- and age-specific differences in phenotypic and molecular characteristics
Source: J Clin Invest. 2024 Jun 11;134(16):e172890. doi: 10.1172/JCI172890 (PMC11324300; doi:10.1172/JCI172890)
Supplement: Supplemental data [file jci-134-172890-s163.pdf]

Supplemental Figures

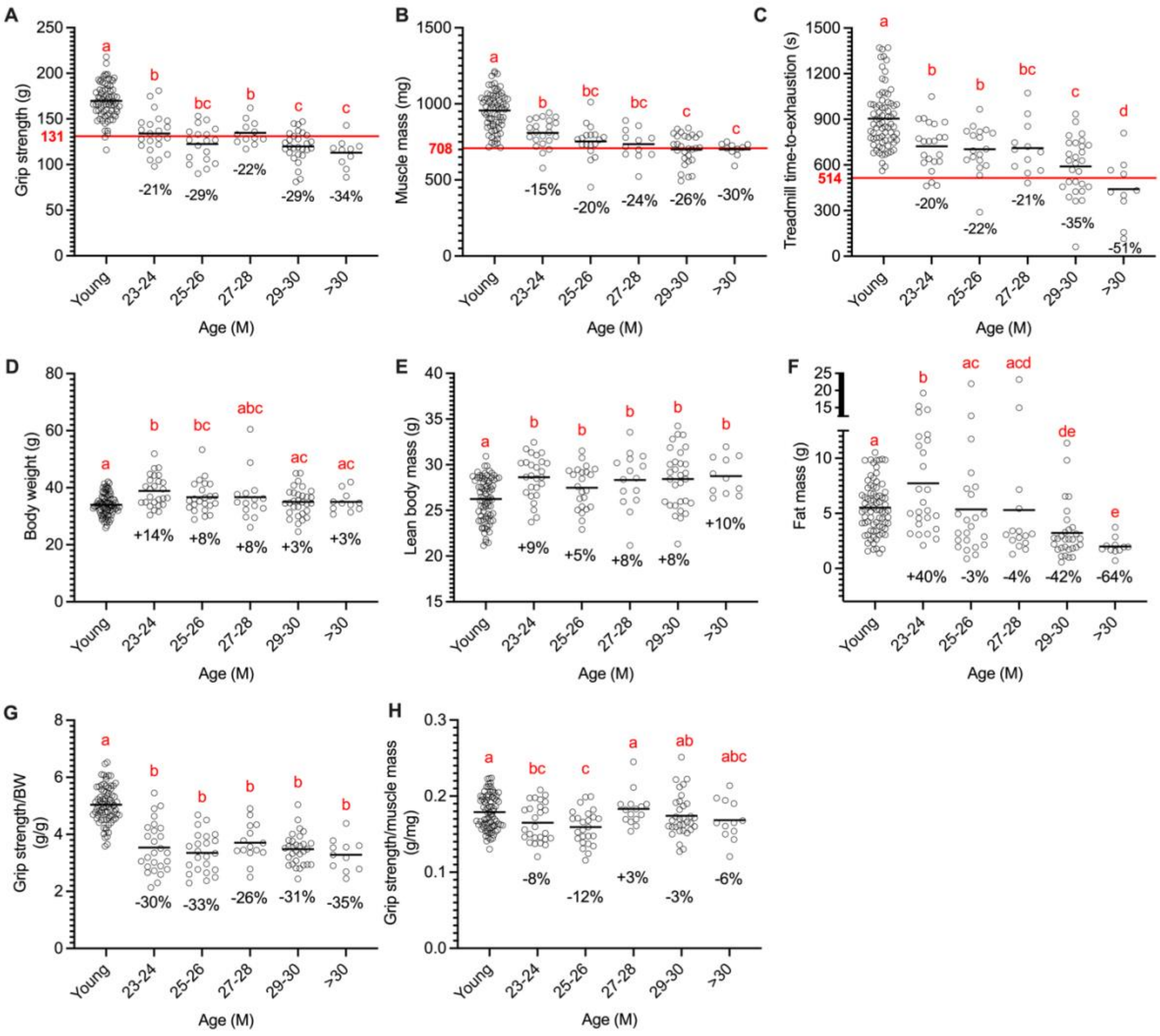

Supplemental Figure 1. Absolute values in sarcopenia measurements, body composition, and normalized grip strength in young (4-9 months) and old (23-32 months) C57BL/6J male mice. (**A**) Forelimb grip strength (g), (**B**) Muscle mass (g), and (**C**) Treadmill time-to-exhaustion (s) by age group. (**A-C**) The horizontal line (in red) at 2 standard deviations below the young group's mean defines the cutoff for impairment. (**D**) Body weight (g). (**E**) Lean body mass (g). (**F**) Fat mass (g). (G**)** Grip strength normalized to body weight (g/g). (**H**) Grip strength normalized to total hindlimb muscle mass (g/mg). One-way ANOVA was performed to detect differences among different age groups followed by LSD post-hoc tests (ANOVA $p < 0.05$). Significant differences were denoted as different letters (a,b,c,d).

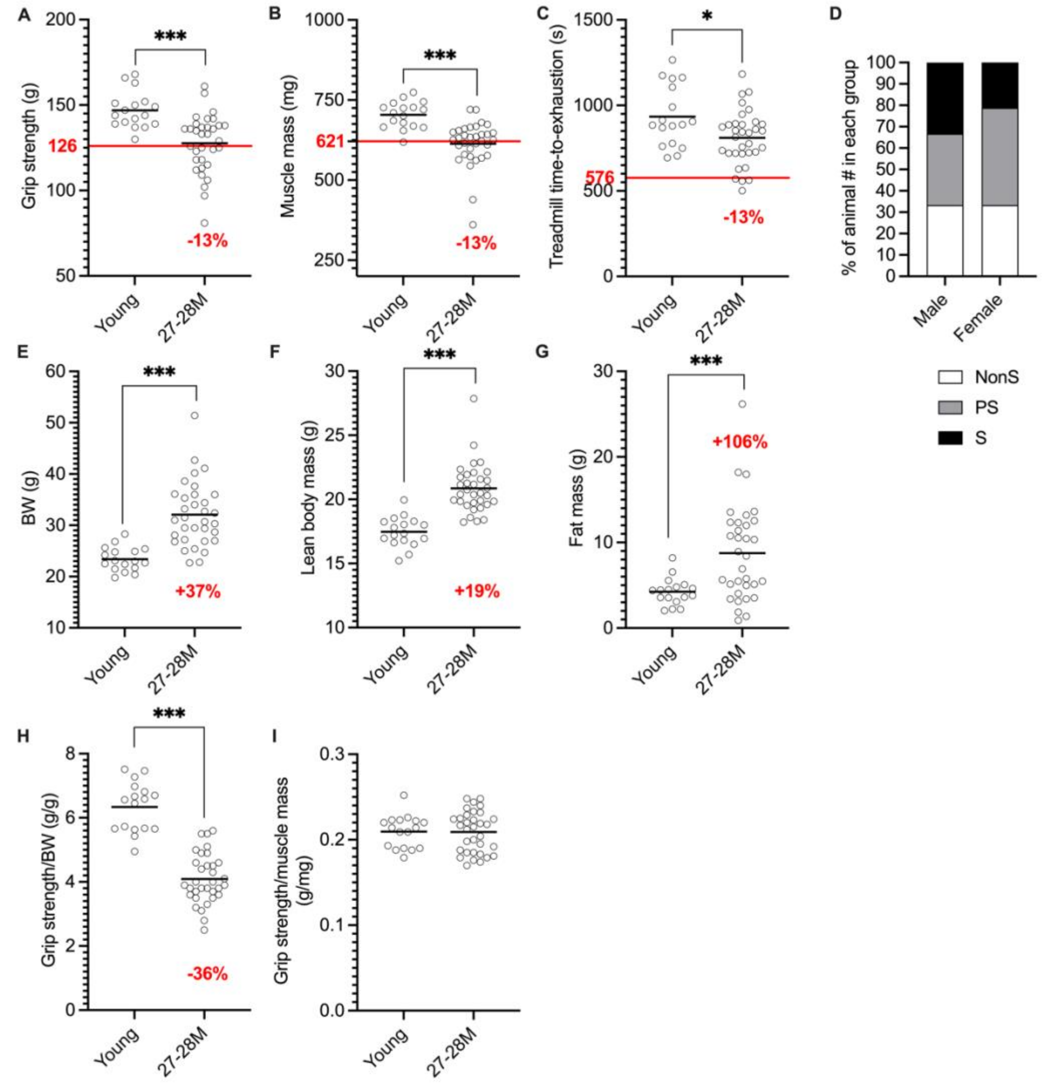

Supplemental Figure 2. Absolute values in sarcopenia measurements, body composition, and normalized grip strength in young (6-7 months) and old (27-28 months) C57BL/6J female mice. (**A**) Forelimb grip strength (g), (**B**) Muscle mass (g), and (**C**) Treadmill time-to-exhaustion (s) by age group. (**A-C**) The horizontal line (in red) at 2 standard deviations below the young group's mean defines the cutoff for impairment. (**D**) Percentage of animals in the age group of 27-28 months (N = 12 for males and 33 for females), is identified by their sarcopenia status as non-sarcopenic (Non-S; 0 deficit), probably sarcopenic (PS, 1 deficit), or sarcopenic (S, 2-3 deficits) based on the numbers of deficits in grip strength, muscle mass, and treadmill running time. (**E**) Body weight (g). (**F**) Lean body mass (g). (**G**) Fat mass (g). (**H**) Grip strength normalized to body weight (g/g). (**I**) Grip strength normalized to total hindlimb muscle mass (g/mg). N = 17 and 33 for young and old females. A two-tailed independent t-test was performed to compare age differences (* $p < 0.05$, ** $p < 0.01$, *** $p < 0.001$).

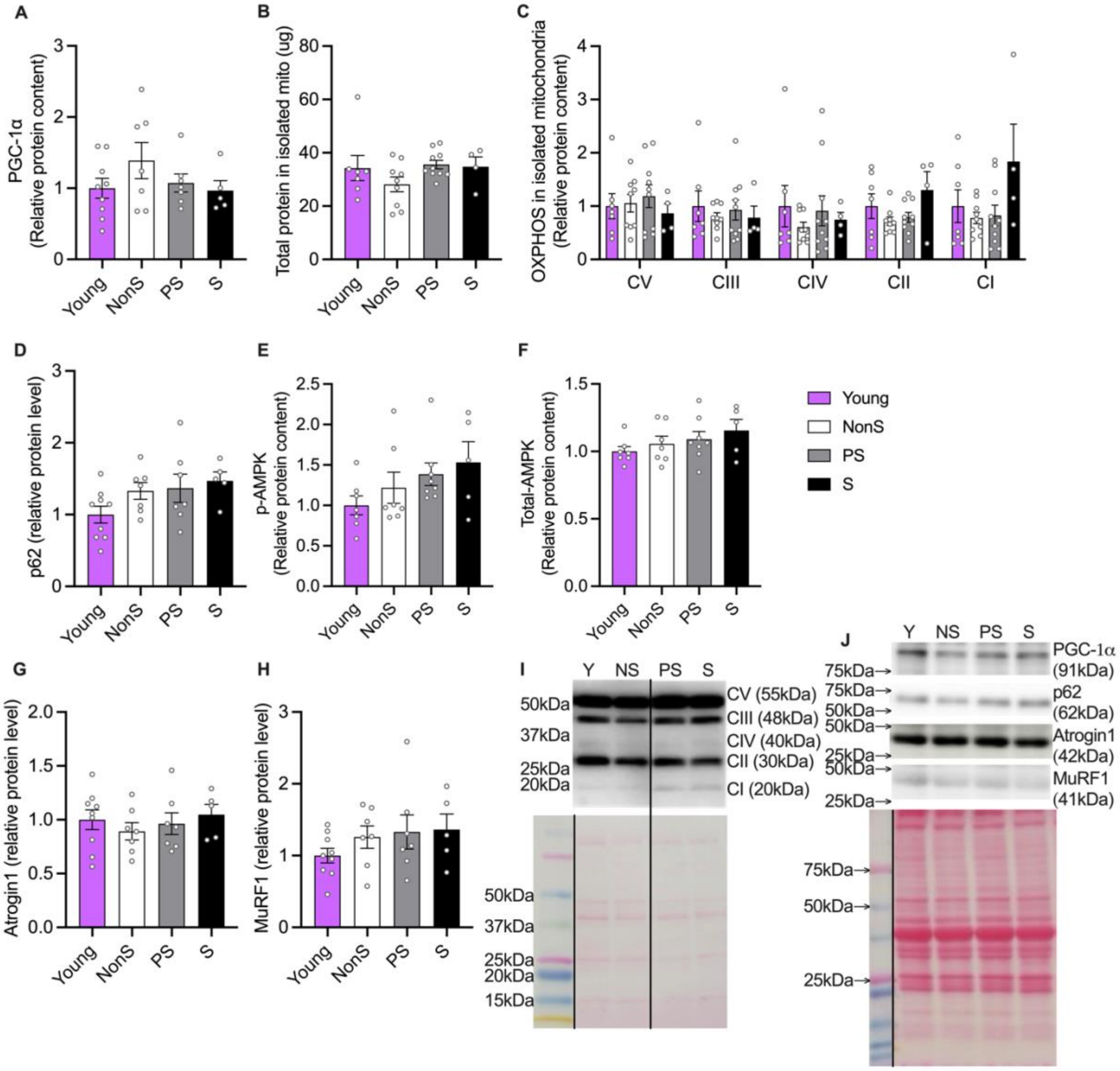

Supplemental Figure 3. Protein levels of mitochondrial markers, autophagy, AMPK signaling and atrogenes in female mice with the progression of sarcopenia. (**A**) Relative protein content of mitochondrial biogenesis marker PGC-1α in GAS muscles (N = 9, 7, 7, 5). (**B**) Total mitochondrial protein in PL muscles detected by BCA (ug, N = 7, 9, 10, 4). (**C**) Relative protein content of OXPHOS complexes in isolated mitochondria from PL muscles (N = 7, 9, 10, 4). (**D**) Relative protein content of mitophagy marker p62 in GAS (N = 9, 7, 7, 5). Protein levels of (**E**) p-AMPKα (Thr172) and (**F**) total AMPK in Quad muscles detected by MSD electrochemiluminescence immunoassay (N = 7, 7, 8, 5). Relative protein content of atrogenes (**G**) Atrogin1, and (**H**) MuRF1 in GAS muscles (N = 9, 7, 7, 5). Representative Western blots and Ponceau red staining for (**I**) OXPHOS complexes and (**J**) PGC-1α, p62, Atrogin1, and MuRF1. Data are shown as mean ± SE. One-way ANOVA was used to identify differences across groups. Since none of the ANOVA tests for these parameters yielded significant results (ANOVA $p > 0.1$), Tukey's HSD post-hoc tests were subsequently conducted. OXPHOS complexes and p-AMPKα protein levels were analyzed by Kruskal-Wallis tests as the data was not normally distributed ($p < 0.05$).

Supplemental Methods

Animal experiments

A week before the experiments, mice were acclimated to their cages and human handling. Treadmill tests, grip tests, and body composition were evaluated the week before termination. Body composition, including body weight, lean body mass (LBM) and fat mass (FM), was measured by nuclear magnetic resonance (NMR, Bruker Optics, The Woodlands, TX, USA) while the mouse was conscious. Mice were euthanized by either a CO_2-infused chamber or isoflurane followed by cervical dislocation. Hindlimb muscles were collected and weighed for measuring muscle mass and for biochemical analysis, including soleus, plantaris (PL), gastrocnemius (GAS), extensor digitorum longus (EDL), tibialis anterior (TA), and quadriceps (Quad). A subset of mice used for muscle physiology or mitochondrial respiration assay was euthanized via cervical dislocation while under isoflurane-induced anesthesia.

Treadmill test

The Exer-6 M treadmill was used for treadmill tests (Columbus Instruments, Columbus, OH). Before the treadmill test, mice were placed on the static treadmill for five minutes for acclimation. The protocol started at a speed of 5 meters (m)/minute. Following these 5 minutes, speed was increased by 1 m every minute. The mouse was motivated to run by lightly tapping at the bottom using a cotton swab until exhausted. If, after three attempts to encourage the mouse to run by tapping its bottom, the animal still refuses to run and remains near the tester at the end of the treadmill, the mouse is considered exhausted, and the test is concluded. The protocol and volitional fatigue criterion were both adapted from Kwak et al. (1). The total amount of time (seconds) the mouse remained on the treadmill was recorded.

Grip strength

Grip strength was measured right before euthanasia by a grip strength meter with a digital force gauge (Columbus Instruments, Columbus, OH). Prior to grip strength testing, mice were placed on the gripping grid to help them become accustomed to the environment without applying any pulling force. Forelimb grip strength was assessed by allowing the mouse to grasp a pull bar connected to a force gauge by only using its forelimbs. The maximum grip strength was recorded on the force gauge in kilograms. The test was performed three times at one-minute intervals. Maximum grip strength was recorded. Absolute grip strength (g), and grip strength normalized to body weight (g/g) or hindlimb muscle mass (g/g) were reported.

In situ muscle physiology

A muscle physiology test was performed on TA muscles in situ using the Aurora 1300A 3-in-1 Whole Animal System for Mice (Aurora Scientific, Ontario, Canada). The procedure was adapted from Rich Lovering's protocol (SOP#: MDC1A_M.2.2.002) and Gordon S. Lynch's protocol (SOP#: DMD_M.2.2.005). Before the terminal surgery, mice were anesthetized with 2% isoflurane via a nose cone and placed on a heated platform. The distal tendon of the left TA was isolated and tied to a force transducer using a 4-0 suture (Fine Scientific Tools). The TA muscle was then stimulated by a pair of needle electrodes placed directly on the deep side of the muscle's proximal end. Optimal length (Lo, mm) was identified by a caliper during the single twitch tests. After identifying the Lo, tetanus (150Hz) and force-frequency tests (10, 30, 50, 75, 100, 125, 150, 200Hz) were performed at one-minute intervals for each stimulation. At the end of the experiment, the mouse was euthanized by cervical dislocation while still under anesthesia, and the TA muscle was collected for muscle weight evaluation. Peak

force (Po) was evaluated as the highest force production during the force-frequency test. The physiological CSA (pCSA) was identified using the following equation: pCSA (mm^2) = muscle mass (mg) / [1.06 x Lo (mm) x 0.6], where 1.06 is the skeletal muscle density and 0.6 is the TA length-to-fiber ratio. Both values were established from previous studies (2, 3). Specific force (SPo) was calculated as peak force divided by the physiological CSA.

Immunohistochemistry and succinate dehydrogenase (SDH) staining

The CSA of individual fibers from the PL muscle was determined as previously described (4-6). Briefly, the OCT-mounted GAS/PL muscle was sliced at 10 µm using a Cryostat (Leica CM3050S, Nußloch, Germany) at -22ºC. All muscles were transected at the mid-belly area, the largest cross-section of the whole muscle. The muscle sections were dehydrated for 30 minutes and blocked with 10% goat serum in Phosphate Buffered Saline (PBS) for one hour. Primary antibodies were applied for two hours at room temperature. Primary antibodies and dilutions were used as follows: BA-D5 (1:10), which detects myosin heavy chain (MHC)-I fibers; SC-71 (1:300), which detects MHC-IIA fibers; BF-F3 (1:50), which detects type MHC-IIB fibers (Developmental Studies Hybridoma Bank, Iowa City, IA), and anti-dystrophin for detecting membranes (1:100, ab15277, Abcam). After three washes in PBS, sections were incubated in the corresponding secondary antibodies (Thermo Fisher Scientific, Waltham, MA) for 1 hour: Goat anti-Mouse IgG2b, Alexa Fluor™ 350 (A21140, 1:500) for MHC-I; Goat anti-Mouse IgG1, Alexa Fluor™ 488 (A21121, 1:500) for MHC-IIA; Goat anti-Mouse IgM (Heavy chain), Alexa Fluor™ 555 (A21426, 1:500) for MHC-IIB; and Goat anti-Rabbit IgG (H+L), Alexa Fluor™ Plus 647 (A32733, 1:200) for membranes. After another three washes in PBS, the sections were mounted with Prolong Gold AntiFade reagent (P36934, Thermo Fisher Scientific).

The 10x PL muscle cross-sectional image was obtained using a Nikon Ni-E microscope and NIS-Elements software (Nikon, Tokyo, Japan). To analyze the CSA of muscle fibers, approximately 100 type IIA, 200 type IIB, and 60-80 type IIX or IIA/X fibers from the whole PL area were analyzed using the same methods. The percentage of each fiber type in PL muscles was analyzed by using the Cell Counter plugin from Image J analysis software (National Institutes of Health, http://rsb.info.nih.gov/ij/).

The procedure of SDH staining protocol has been previously described (7). The sections of skeletal muscles were first incubated with a mixed solution containing 10 ml of 0.2 M Phosphate Buffer (pH 7.6), composed of 0.2 M NaH2PO4 and 0.2 M Na2HPO4, along with 270 mg of sodium succinate (S2378, Sigma) and 10 mg of nitro blue tetrazolium (NBT, N6876, Sigma) for 30 minutes at 37°C. After that, sections were washed three times with dH2O to remove any unbound NBT. Then, three exchanges of acetone solutions in increasing concentrations (30%, 60%, and 90%) were performed to wash off any remaining unbound NBT. The sections were then rinsed several times with dH2O and finally mounted with an aqueous mounting medium. To quantify the SDH-positive area in PL muscles, the PL area was identified using the free-hand tool in ImageJ (Fiji). A standard threshold (Min = 20; Max = 254) was then applied to all images to quantify the SDH-positive area. The final percentage of SDH-positive area was calculated as the SDH-positive area/ total PL area. All procedures were conducted by three trained and blinded researchers using the ImageJ (Fiji) analysis software (National Institutes of Health, http://rsb.info.nih.gov/ij/).

Real-time reverse transcription-quantitative polymerase chain reaction (RT-PCR)

Half of the GAS/PL muscle from the left side of the animal was saved in RNAlater® (76104, Qiagen, Valencia, CA) after harvesting. RNA was isolated using the Qiagen RNeasy mini kit (74104, Qiagen). Transcription levels of the isolated RNA were identified by BioTek Cytation 5. Total RNA was reverse transcribed to cDNA by QuantiTect Reverse Transcription Kit (205311, Qiagen). RT-PCR was detected by an ABI 7500 instrument (Applied Biosystems, Foster City, CA) by using predesigned TaqMan Expression Assays (Thermo Fisher

Scientific, Waltham, MA). The quantification of genes of interest was normalized to a reference gene hypoxanthine guanine phosphoribosyl transferase (*Hprt*) and expressed as a relative fold-change of the young group by a standard 2-ΔCT method. The following TaqMan primers from Thermo Fisher Scientific (4331182) were used in this study: *Sqstm1* and *Map1lc3a*.

Western Blotting

Western blotting was performed to identify the protein content of peroxisome proliferator-activated receptor-gamma coactivator (PGC-1α), p62, Atrogin-1, and MuRF1 in GAS/PL muscles and oxidative phosphorylation (OXPHOS) complexes in PL muscles. A portion of GAS/PL muscle (~60 mg) was homogenized in Cellytic™ Mt Cell Lysis Reagent (C3228, Sigma) buffer with a cocktail of protease and phosphatase inhibitors (11697498001 and 4906845001, Roche). The homogenate was centrifuged at 10,000 g for 15 minutes at 4°C, and the supernatant was collected for protein assays. For OXPHOS content, isolated mitochondria were used for Western blotting (see "Mitochondria isolation and mitochondrial respiration measurements"). The protein concentration was further quantified by a bicinchoninic acid (BCA) assay (23227, Thermo Fisher Scientific) using bovine serum albumin (BSA) as a standard. Prior to electrophoresis, protein extractions were diluted with 5x Lane Marker Reducing Sample Buffer (PI39000, Thermo Fisher Scientific) and heated at 95°C for 4 minutes. 50 µg protein was loaded onto 4–15% Criterion™ TGX™ Precast Midi Protein Gels (5671084, BIO-RAD, Hercules, CA) and separated by using a BIO-RAD Criterion™ Cell electrophoresis system (165-6001). Kaleidoscope Prestained Protein Standards (Bio-Rad, 1610375) or Precision Plus Protein Dual Color Standards (Bio-Rad, 1610374) were loaded at both ends of the membrane to visualize the levels of various molecular weights. The proteins were then transferred to nitrocellulose membranes by a BIO-RAD Criterion™ Blotter (170-4070) at 100 V, 4°C, for 30 minutes. The membranes were stained with Ponceau S Staining Solution (59803, Cell signaling) and then washed off by TBS/T after imaging. After transferring, we used the Kaleidoscope (161-0375) or Dual Color (161-0374) standards and Ponceau red stain as guidance to cut the membrane based on molecular weight precisely. This resulted in clear demarcations at 25 kDa, 50 kDa, and 75 kDa. The Ponceau red stain was then washed off with TBS/T before proceeding to the blocking step. After blocking in 5% non-fat dry milk in TBS/T at room temperature for 1 hour, membranes were incubated with primary antibodies overnight at 4°C. The following primary antibodies and dilutions were used for the experiments: anti-SQSTM1/p62 antibody (1:1000. Ab56416, Abcam, Cambridge, MA); Anti-PGC1 alpha antibody (1:1000. ab191838, Abcam); Total OXPHOS Rodent WB Antibody Cocktail (1:1000. ab110413, Abcam); anti-Atrogin antibody (1:1000. Thermo Fisher, PA5-19056); anti-Murf1 antibody (1:2000. R&D Systems, AF5366). After incubation, the membranes were washed and probed with the corresponding HRP-conjugated secondary antibodies. The membranes were developed using SuperSignal™ West Dura Extended Duration Substrate (34075, Thermo Fisher Scientific) and imaged by ImageQuant LAS 4000 (GE Health Care, Chicago, IL). The quantification of densitometry was analyzed by ImageJ and expressed as a ratio over the Ponceau red signal.

Electrochemiluminescence immunoassay

Total AMPK and p-AMPK (Thr172) in Quad were detected by MULTI-ARRAY 96 Plate Pack, SECTOR Plate (Meso-Scale Diagnostics, MSD, L15XA-3, Rockville, MD). The procedure was adapted from an established protocol (8) and described previously (9). In brief, each plate was prepared by coating with 200 ng/well anti-AMPK alpha 1 + AMPK alpha 2 antibody [34.2] (ab80039, Abcam) at 4°C overnight. On the following day, the plate was washed with 1x MSD Tris wash buffer followed by incubating with 1% MSD Blocker A (R93BA-4, MSD) for 1 hour. After one more wash, 125 µg protein lysate was loaded and incubated at 37°C for 2 hours. After three washes, 25 ng/well rabbit anti-phospho-AMPKα (Thr172, Cell Signaling, 2535) or 100 ng/well rabbit anti-total AMPK (Cell Signaling, 5831) antibody was added and incubated for 1.5 hours at 37°C. After one wash, 50 ng

MSD anti-rabbit SULFO-TAG antibody (R32AB) was added per well and incubated for 1 hour at 37°C. After another three washes, 2x MSD Read buffer diluted from MSD Read Buffer T (4x, R92TC-3, MSD) was added and the plate was read on MSD Sector Imager (MSD).

Mitochondria isolation and mitochondrial respiration measurements

The mitochondria isolation method is adapted from an established protocol published previously (10). Plantaris muscles from both sides were harvested while the mouse was under deep anesthesia by isoflurane and immediately saved in mitochondria isolation buffer ((MIB), 210 mM sucrose, 2 mM EGTA, 40 mM NaCl, 30 mM HEPES, pH 7.4) on ice. The muscle was then homogenized in MIB by Kimble homogenizer and centrifuged at 900g and 4°C for 10 minutes. The supernatant was collected and centrifuged again at 10,000g and 4°C for 10 minutes. The mitochondrial pellet was collected and resuspended in MIB, and protein concentration was identified by Pierce Rapid Gold BCA Protein Assay (A53227, Thermo Fisher Scientific). After another centrifuge at 10,000g and 4°C for 10 minutes, isolated mitochondria were resuspended in mitochondrial assay solution ((MAS), 70 mM sucrose, 220 mM d-mannitol, 10 mM KH2PO4, 5 mM MgCl2, 2 mM HEPES, 1 mM EGTA, 0.2% fatty-acid free BSA, pH 7.4) with substrates (5 mM malate and 5 mM pyruvate).

Mitochondrial respiration was detected by Agilent Seahorse XFe24 Analyzer (Agilent Technologies, Santa Clara, CA) by using the methods adapted from Rodger's protocol (11, 12) and was described previously (7). The isolated mitochondria in MAS with substrates were plated in Agilent Seahorse XF24 Cell Culture Microplates (7.5 µg/well in triplicates). 50 ul of adenosine diphosphate (ADP, 4 mM), 55ul oligomycin (2 uM), 60ul carbonyl cyanide-p-trifluoromethoxy-phenylhydrazone (FCCP, 4 uM), and 65ul of antimycin A (2 uM) were loaded into the cartridge plate and injected into the cell plate in sequence. Oxygen consumption rate ((OCR), pmol/minutes) of each respiration state was measured using an XFe24 Seahorse Analyzer (Agilent Technologies) in real-time.

Abundance Proteomics

Batch design, sample lysis and digestion

The samples were divided into two batches, each containing 12 individual samples and two pooled references, for a total of 14 samples per batch. A pool of all 24 samples was used to create a reference pool to be used as a common reference, which was homogenized, aliquoted, frozen, and used to compare the two batches. Six young (6-7 months) and six old (27-29 months) male mice in the current study were included in the aforementioned batch design with additional samples utilized for a separate study.

Large chunks of mouse gastrocnemius muscle tissue (30 mg to 100 mg) were first homogenized in 5% sodium dodecyl sulfate (SDS)/50 mM triethylammonium bicarbonate (TEAB) lysis buffer (Sigma) with protease and phosphatase inhibitors in a Bullet Blender Storm (Next Advance, Inc) with 0.5 mm zirconium oxide beads at 4°C. Buffer volume was added to 5 times tissue weight and bead volume was 3 times tissue weight. The homogenate was then further lysed with a probe sonicator (Fisher Scientific) for 3 cycles of setting #3 for 2 minutes on ice in between lysis steps. Protein concentration was measured using the Pierce BCA assay (Thermo Scientific). A homogenate of 50 µg was added to a process control containing 800 ng of yeast enolase protein (Sigma), then reduced with 20 mM DTT and alkylated with 40 mM IAA. Lysates were prepared for S-trap column (Protifi) binding by the addition of 1.2% phosphoric acid and 350 µl of binding buffer (90% Methanol, 100 mM TEAB). The acidified lysate was incrementally bound to the column, followed by three wash steps with binding buffer to remove SDS, three wash steps with a 50:50 methanol:chloroform mixture to remove lipids, and a final wash step with binding buffer. Trypsin (1:10) in 50mM TEAB was added to the S-trap column for digestion at 47°C for one

hour. Peptides were eluted with increasing hydrophobicity with a first elution of 50 mM TEAB, followed by elutions with 0.1% trifluoroacetic acid (TFA) and then 50% acetonitrile in water. Elutions were pooled, speed vacuumed and resuspended in 0.1% TFA.

Liquid chromatography and DIA mass spectrometry

Peptides were eluted from a PepSep C18 15 cm column with 150 µm inner diameter and 1.9 µm particle size (Bruker), combined with a PepMap C18 5 mm Neo Trap Cartridge with 300 µm inner diameter (Thermo Scientific), and a Fossil Sharp Singularity LOTUS 5 cm hydrophobic coated nESI emitter with 20 µm inner diameter and 363um outer diameter (Fossil Ion Technology). A 50°C heated source (CorSolutions) was used to electrospray two µg of each digested sample with 300 femtomole of Pierce Retention Time Calibrant (PRTC) onto a Thermo Scientific Vanquish Neo UHPLC system coupled with a Thermo Orbitrap Eclipse Tribrid Mass Spectrometer with the application of a distal 3 kV spray voltage.

The PRTC was used to assess the system suitability of the column and instruments before and during analysis. We analyze system suitability runs prior to any sample analysis and then after every six to eight sample runs another system suitability run is analyzed. Buffer A is 0.1% formic acid in water and buffer B is 0.1% formic acid in 80% acetonitrile.

The 25-minute system suitability gradient consists of a 4 to 6% B in 0.7 minutes, 6 to 6.5% B in 0.3 minutes, 6.5 to 40% B in 20 minutes, 40 to 55% B in 0.5 minutes, followed by a wash of 99% B for 3.5 minutes at a flow rate of 1.3 uL/minutes. The 60-minutesute sample LC gradient consists of a 4 to 6% B for 0.7 minutes, 6 to 6.5% B in 0.3 minutes, 6.5 to 40% B in 55.7 minutes, 40 to 55% B in 0.5 minutes, followed by a wash of 99% B for 2.8 minutes at a flow rate of 0.8 to 1.3 uL/minutes.

For the system suitability analysis, a cycle of one 30,000 resolution full-scan mass spectrum (400-810 *m/z*) with AGC target of 4e5 and maximum injection time of 50 millisecond (ms) followed by a data-independent acquisition (DIA) MS/MS spectra on a loop count of 20 using an inclusion list of +2 precursor charges at 15,000 resolution, AGC target of 5e4, 22 ms maximum injection time, 30% normalized collision energy with a 2 *m/z* isolation window.

For the sample digest, first a chromatogram library of 6 independent injections is analyzed from a pool of all samples within a batch. For each injection a cycle of one 30,000 resolution full-scan mass spectrum with AGC target of 4e5 and maximum injection time of 50 ms and a mass range of 110 *m/z* (395-505 *m/z*, 495-605 *m/z*, 595-705 *m/z*, 695-805 *m/z*, 795-905 *m/z*, 895-1005 *m/z*) followed by a DIA MS/MS spectra on a loop count of 25 using an inclusion list of +3 precursor charges at 30,000 resolution, AGC target of 5e5, 54 ms maximum injection time, 27% normalized collision energy with a 4 *m/z* overlapping isolation window. The chromatogram library data is used to quantify proteins from individual sample runs.

These individual runs consist of a cycle of one 30,000 resolution full-scan mass spectrum with a mass range of 350-1005 *m/z* with AGC target of 4e5 and maximum injection time of 50 ms followed by a DIA MS/MS spectra on a loop count of 75 using an inclusion list of +3 precursor charges at 30,000 resolution, AGC target of 4e5, 54 ms maximum injection time, 27% normalized collision energy with an overlapping 12 *m/z* isolation window. Application of the mass spectrometer and LC solvent gradients are controlled by the ThermoFisher Xcalibur data system.

DIA signal processing

System suitability runs and process controls are analyzed using Skyline and AutoQC via PanoramaWeb (13, 14). Thermo XCalibur RAW files are converted to mzML format using Proteowizard using vendor peak picking and

demultiplexing with the settings of “overlap_only” and Mass Error = 10.0 ppm (15). On column chromatogram libraries are created using the data from the six gas phase fractionated “narrow window” DIA runs of the pooled samples from each batch (16). These narrow windows are analyzed using EncyclopeDIA with the default settings (10 ppm tolerances, trypsin digestion, HCD b- and y-ions) of a Prosit predicted spectra library based on the Uniprot mouse canonical FASTA (17-19). The results from this analysis are saved as a “Chromatogram Library" in EncyclopeDIA’s eLib format where the predicted intensities and iRT of the Prosit library are replaced with the empirically measured intensities and RT from the gas phase fractionated LC-MS/MS data. The “wide window” DIA runs are analyzed using EncyclopeDIA requiring a minimum of 3 quantitative ions and filtering peptides with q-value ≤ 0.01 using Percolator 3.01. After analyzing each file individually, EncyclopeDIA is used to generate a “Quant Report" which stores all the detected peptides, integration boundaries, quantitative transitions, and statistical metrics from all runs in an eLib format. The Quant Report eLib library is imported into Skyline using the mouse uniprot FASTA as the background proteome to map peptides to protein groups, perform peak integration, manual evaluation, and report generation. A csv file of peptide level total area fragments (TAFs) for each replicate was exported from Skyline using the custom reporting capabilities of the document grid (20, 21).

Data analysis

A competitive gene set test in young and old mice individually was performed using the ‘camera’ function (22) from the Bioconductor limma package, based on any Kyoto Encyclopedia of Genes and Genomes (KEGG) pathway that had 10 or more proteins represented in this analysis. The camera function uses statistics from any linear model, while accounting for inter-protein correlations, to determine if a given set of proteins is more highly ranked than proteins that are not in the set. We then fitted a linear model for each protein, correlating protein abundance with one of the sarcopenia measures in young and old mice individually and a main effect analysis was performed to identify KEGG pathways that showed similar patterns in young and old mice in terms of correlations between protein abundance and sarcopenia measurements (False discovery rate, FDR < 0.05).

Data availability

The Skyline documents, raw files for quality control and DIA data are available at Panorama Public. ProteomeXchange ID: PXD048723. Access URL: https://panoramaweb.org/mouse-gastroc-sarcopenia-proteomics.url

References for Supplemental Methods:

1. Kwak D, Baumann CW, and Thompson LV. Identifying Characteristics of Frailty in Female Mice Using a Phenotype Assessment Tool. *J Gerontol A Biol Sci Med Sci.* 2020;75(4):640-6.
2. Burkholder TJ, Fingado B, Baron S, and Lieber RL. Relationship between muscle fiber types and sizes and muscle architectural properties in the mouse hindlimb. *J Morphol.* 1994;221(2):177-90.
3. Brooks SV, and Faulkner JA. Contractile properties of skeletal muscles from young, adult and aged mice. *J Physiol.* 1988;404:71-82.
4. Baumann CW, Liu HM, and Thompson LV. Denervation-Induced Activation of the Ubiquitin-Proteasome System Reduces Skeletal Muscle Quantity Not Quality. *PLoS One.* 2016;11(8):e0160839.
5. Liu HM, Ferrington DA, Baumann CW, and Thompson LV. Denervation-Induced Activation of the Standard Proteasome and Immunoproteasome. *PLoS One.* 2016;11(11):e0166831.
6. Sawano S, Komiya Y, Ichitsubo R, Ohkawa Y, Nakamura M, Tatsumi R, et al. A One-Step Immunostaining Method to Visualize Rodent Muscle Fiber Type within a Single Specimen. *PLoS One.* 2016;11(11):e0166080.
7. Liu H, Zang P, Lee, II, Anderson B, Christiani A, Strait-Bodey L, et al. Growth hormone secretagogue receptor-1a mediates ghrelin's effects on attenuating tumour-induced loss of muscle strength but not muscle mass. *J Cachexia Sarcopenia Muscle.* 2021;12(5):1280-95.

8. Esquejo RM, Salatto CT, Delmore J, Albuquerque B, Reyes A, Shi Y, et al. Activation of Liver AMPK with PF-06409577 Corrects NAFLD and Lowers Cholesterol in Rodent and Primate Preclinical Models. *EBioMedicine.* 2018;31:122-32.
9. Kerr HL, Krumm K, Lee, II, Anderson B, Christiani A, Strait L, et al. EXT418, a novel long-acting ghrelin, mitigates Lewis lung carcinoma induced cachexia in mice. *J Cachexia Sarcopenia Muscle.* 2023.
10. Chavez JD, Tang X, Campbell MD, Reyes G, Kramer PA, Stuppard R, et al. Mitochondrial protein interaction landscape of SS-31. *Proc Natl Acad Sci U S A.* 2020;117(26):15363-73.
11. Rogers GW, Brand MD, Petrosyan S, Ashok D, Elorza AA, Ferrick DA, et al. High throughput microplate respiratory measurements using minimal quantities of isolated mitochondria. *PLoS One.* 2011;6(7):e21746.
12. Boutagy NE, Rogers GW, Pyne ES, Ali MM, Hulver MW, and Frisard MI. Using Isolated Mitochondria from Minimal Quantities of Mouse Skeletal Muscle for High throughput Microplate Respiratory Measurements. *J Vis Exp.* 2015(105):e53216.
13. Bereman MS, MacLean B, Tomazela DM, Liebler DC, and MacCoss MJ. The development of selected reaction monitoring methods for targeted proteomics via empirical refinement. *Proteomics.* 2012;12(8):1134-41.
14. Sharma V, Eckels J, Taylor GK, Shulman NJ, Stergachis AB, Joyner SA, et al. Panorama: a targeted proteomics knowledge base. *J Proteome Res.* 2014;13(9):4205-10.
15. Amodei D, Egertson J, MacLean BX, Johnson R, Merrihew GE, Keller A, et al. Improving Precursor Selectivity in Data-Independent Acquisition Using Overlapping Windows. *J Am Soc Mass Spectrom.* 2019;30(4):669-84.
16. Pino LK, Just SC, MacCoss MJ, and Searle BC. Acquiring and Analyzing Data Independent Acquisition Proteomics Experiments without Spectrum Libraries. *Mol Cell Proteomics.* 2020;19(7):1088-103.
17. Egertson JD, Kuehn A, Merrihew GE, Bateman NW, MacLean BX, Ting YS, et al. Multiplexed MS/MS for improved data-independent acquisition. *Nat Methods.* 2013;10(8):744-6.
18. Searle BC, Pino LK, Egertson JD, Ting YS, Lawrence RT, MacLean BX, et al. Chromatogram libraries improve peptide detection and quantification by data independent acquisition mass spectrometry. *Nat Commun.* 2018;9(1):5128.
19. Ting YS, Egertson JD, Bollinger JG, Searle BC, Payne SH, Noble WS, et al. PECAN: library-free peptide detection for data-independent acquisition tandem mass spectrometry data. *Nat Methods.* 2017;14(9):903-8.
20. MacLean B, Tomazela DM, Shulman N, Chambers M, Finney GL, Frewen B, et al. Skyline: an open source document editor for creating and analyzing targeted proteomics experiments. *Bioinformatics.* 2010;26(7):966-8.
21. Pino LK, Searle BC, Bollinger JG, Nunn B, MacLean B, and MacCoss MJ. The Skyline ecosystem: Informatics for quantitative mass spectrometry proteomics. *Mass Spectrom Rev.* 2020;39(3):229-44.
22. Wu D, and Smyth GK. Camera: a competitive gene set test accounting for inter-gene correlation. *Nucleic Acids Res.* 2012;40(17):e133.
