# Supplementary material for: Mouse sarcopenia model reveals sex- and age-specific differences in phenotypic and molecular characteristics
Source: J Clin Invest. 2024 Jun 11;134(16):e172890. doi: 10.1172/JCI172890 (PMC11324300; doi:10.1172/JCI172890)
Supplement: Unedited blot and gel images [file jci-134-172890-s164.pdf]

Western blots

For the following paper:

Mouse Sarcopenia Model Reveals Sex- and Age-Specific Differences in Phenotypic and Molecular Characteristics

Haiming L. Kerr[1], Kora Krumm[1], Barbara Anderson[1], Anthony Christiani[1], Lena Strait[1], Theresa Li[1], Brynn Irwin[1], Siyi Jiang[1], Artur Rybachok[1], Amanda Chen[1], Elizabeth Dacek[1], Lucas Caeiro[1], Gennifer E. Merrihew[2], James W. MacDonald[3], Theo K. Bammler[3], Michael J. MacCoss[2], Jose M. Garcia[1]

[1]Geriatric Research, Education and Clinical Center, Veterans Affairs Puget Sound Health Care System, Seattle, WA 98108, USA; Gerontology and Geriatric Medicine, University of Washington Department of Medicine, Seattle, WA 98195, USA.

[2]Department of Genome Sciences, University of Washington, Seattle, WA 98195, USA

[3]Department of Environmental and Occupational Health Sciences, University of Washington, Seattle, WA 98195, USA

Full unedited blots for Figure 4D
OXPHOS complexes

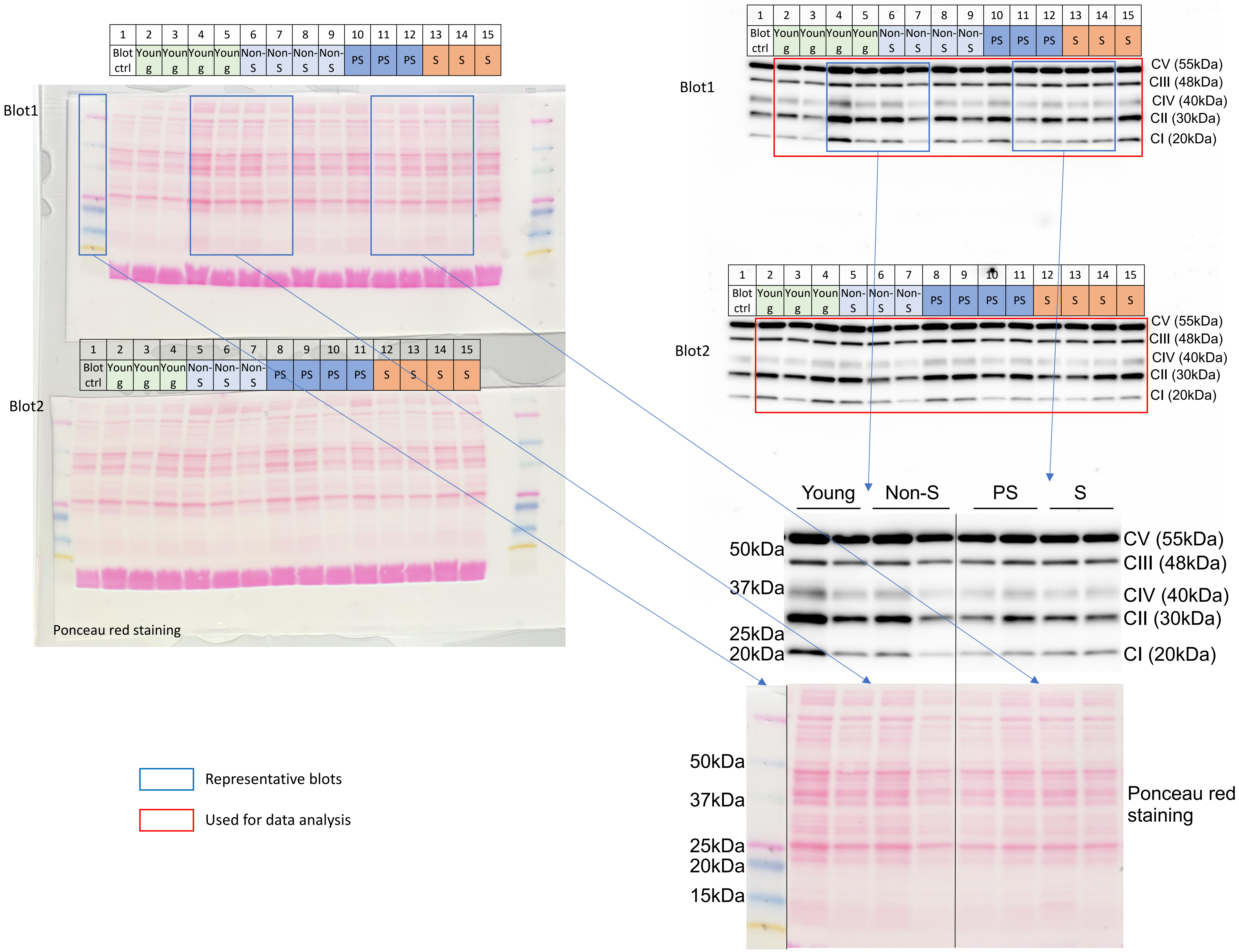

Blots are shown in Fig. 4D

Full unedited blots for Figure 5C
PGC1a blot1

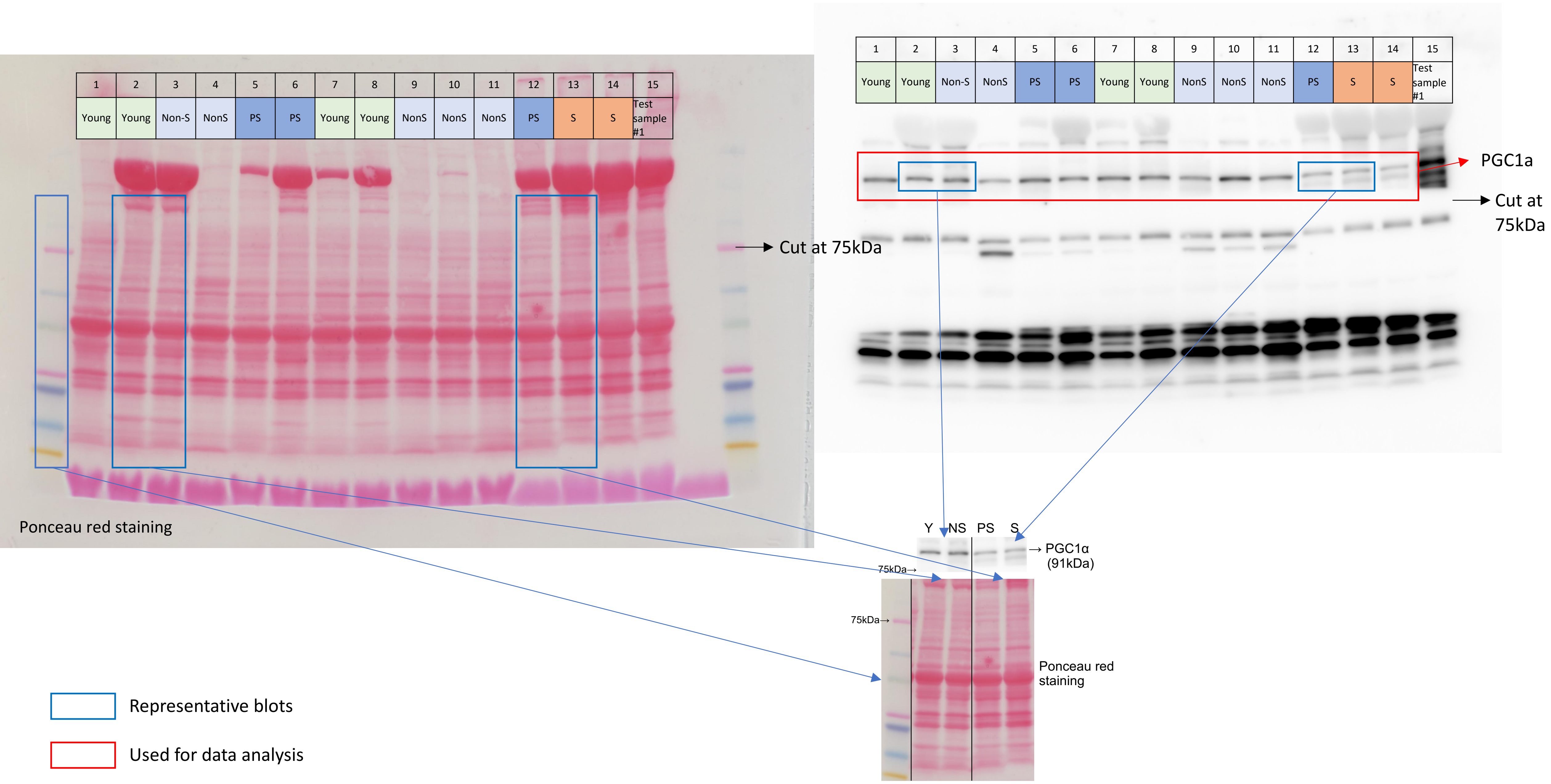

Blots are shown in Fig. 5C

Full unedited blots for Figure 5C
PGC1a blot 2 & 3

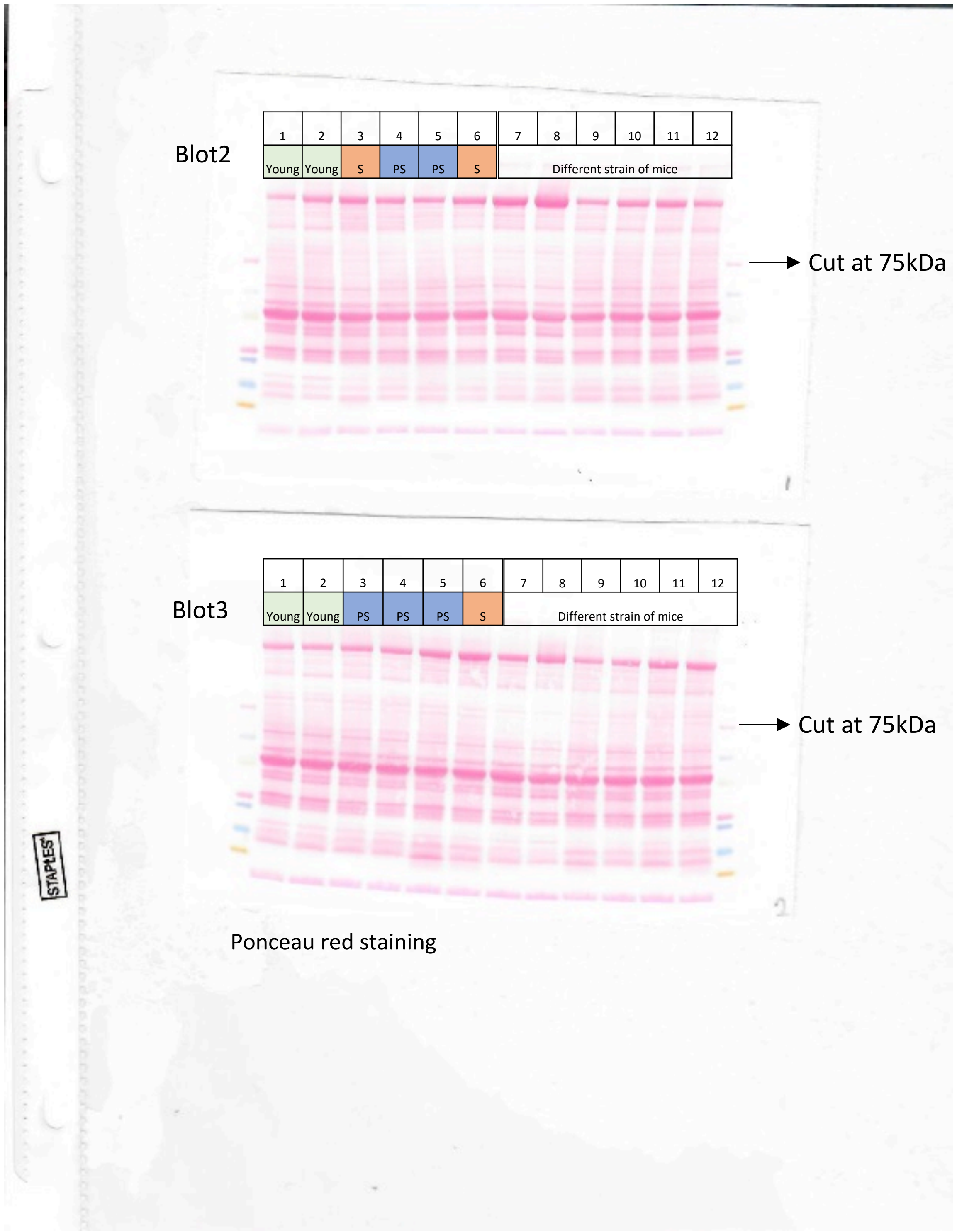

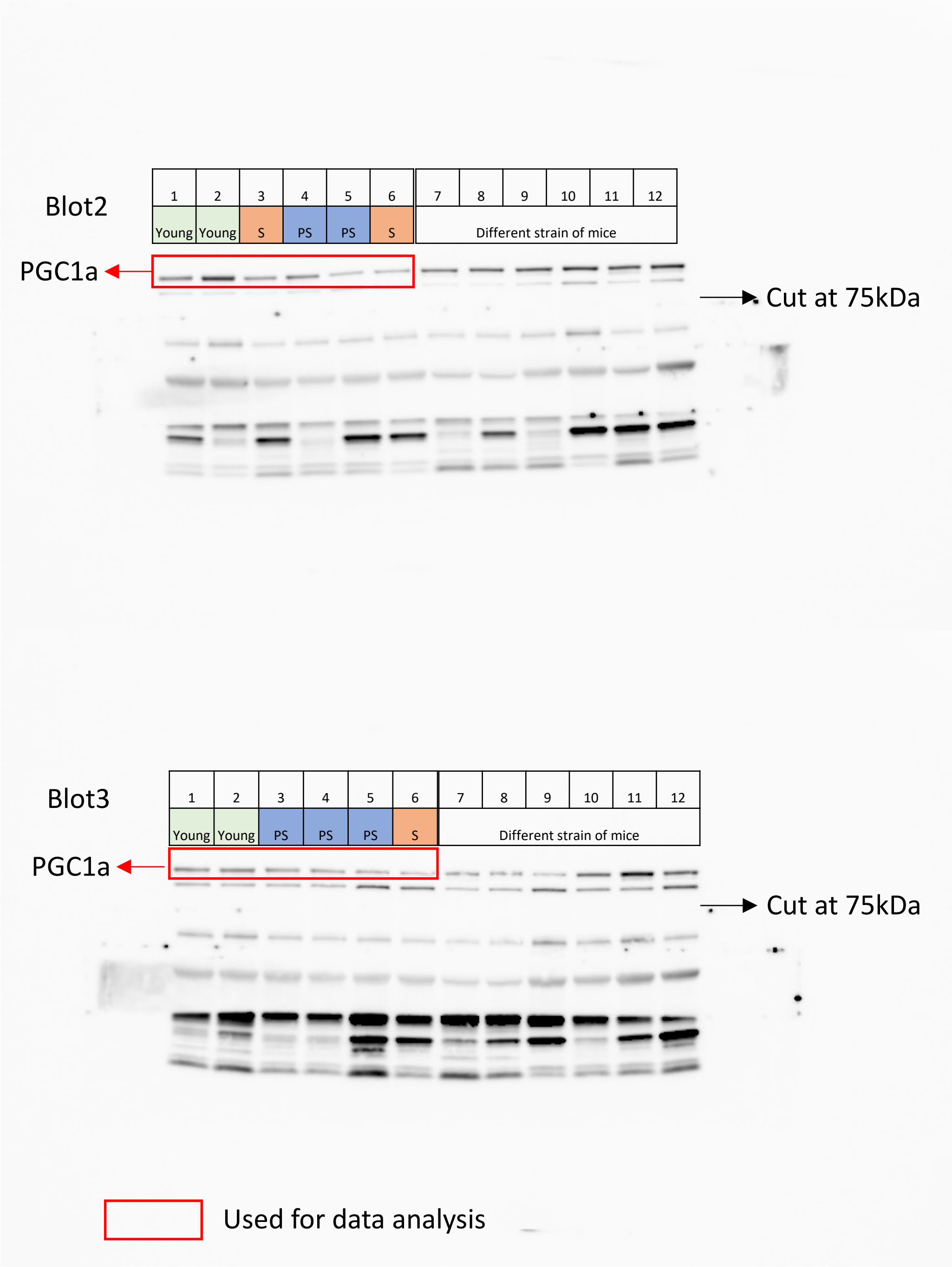

Full unedited blots for Figure 6B
p62 blot 1

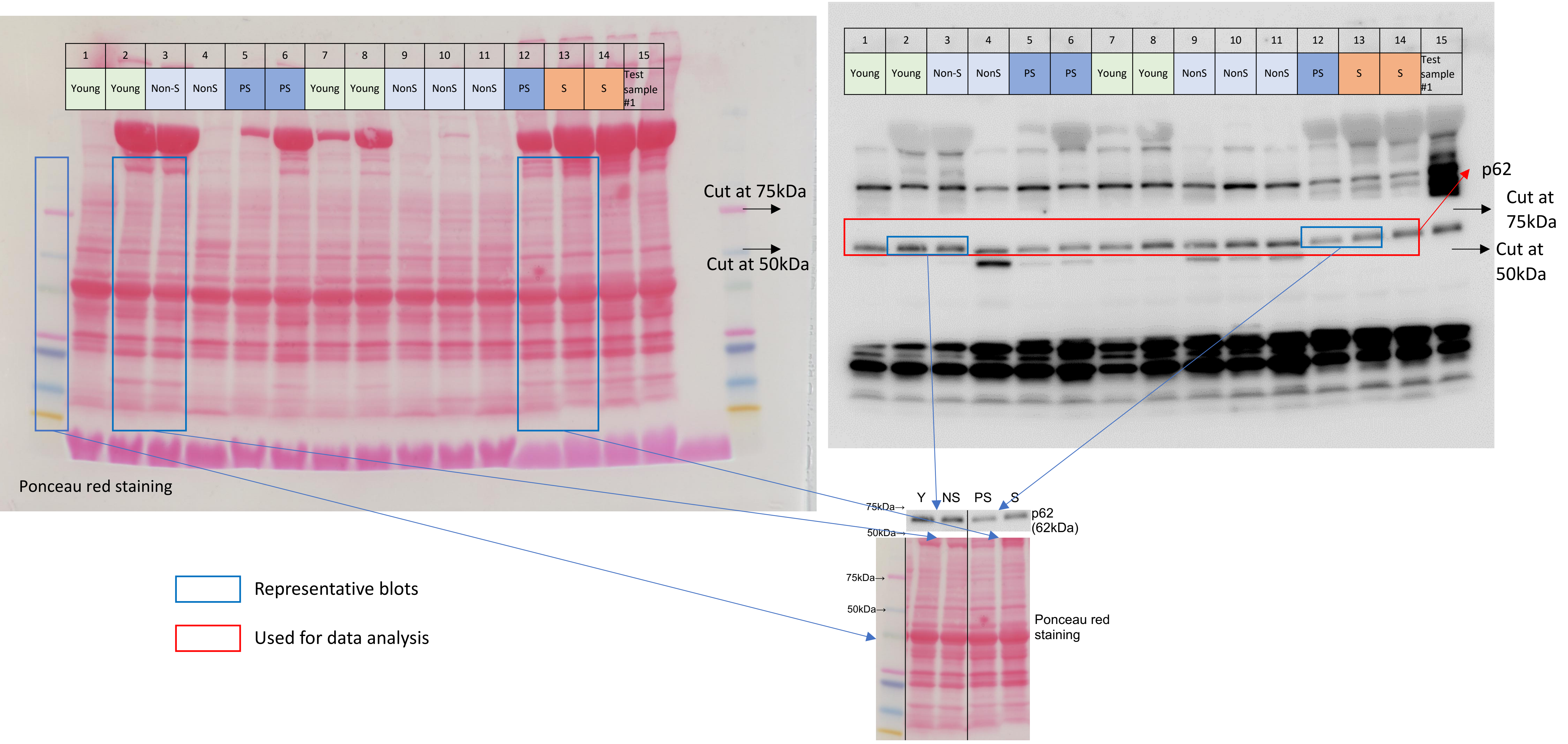

Blots are shown in Fig. 6B

Full unedited blots for Figure 6B
p62 blot 2 & 3

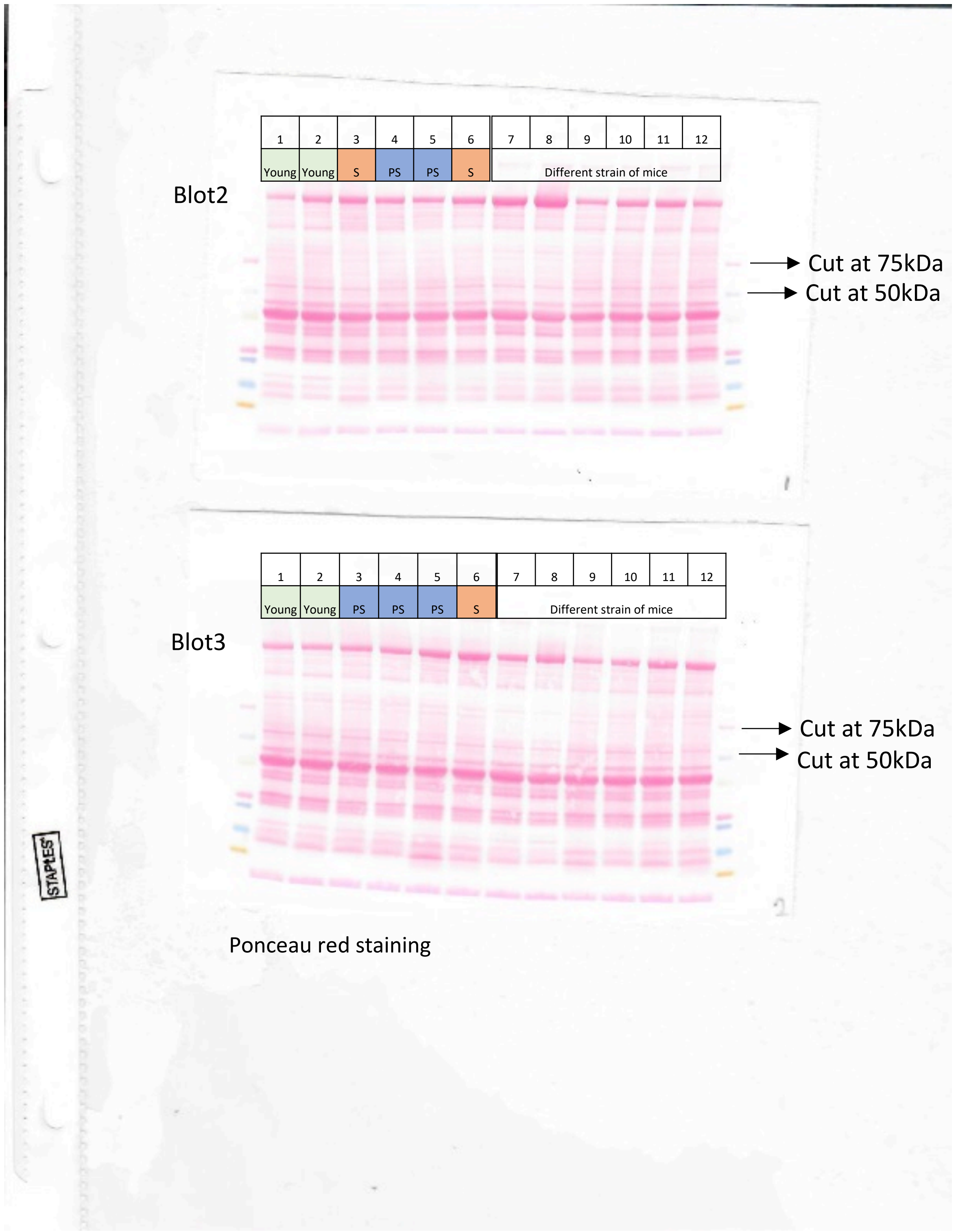

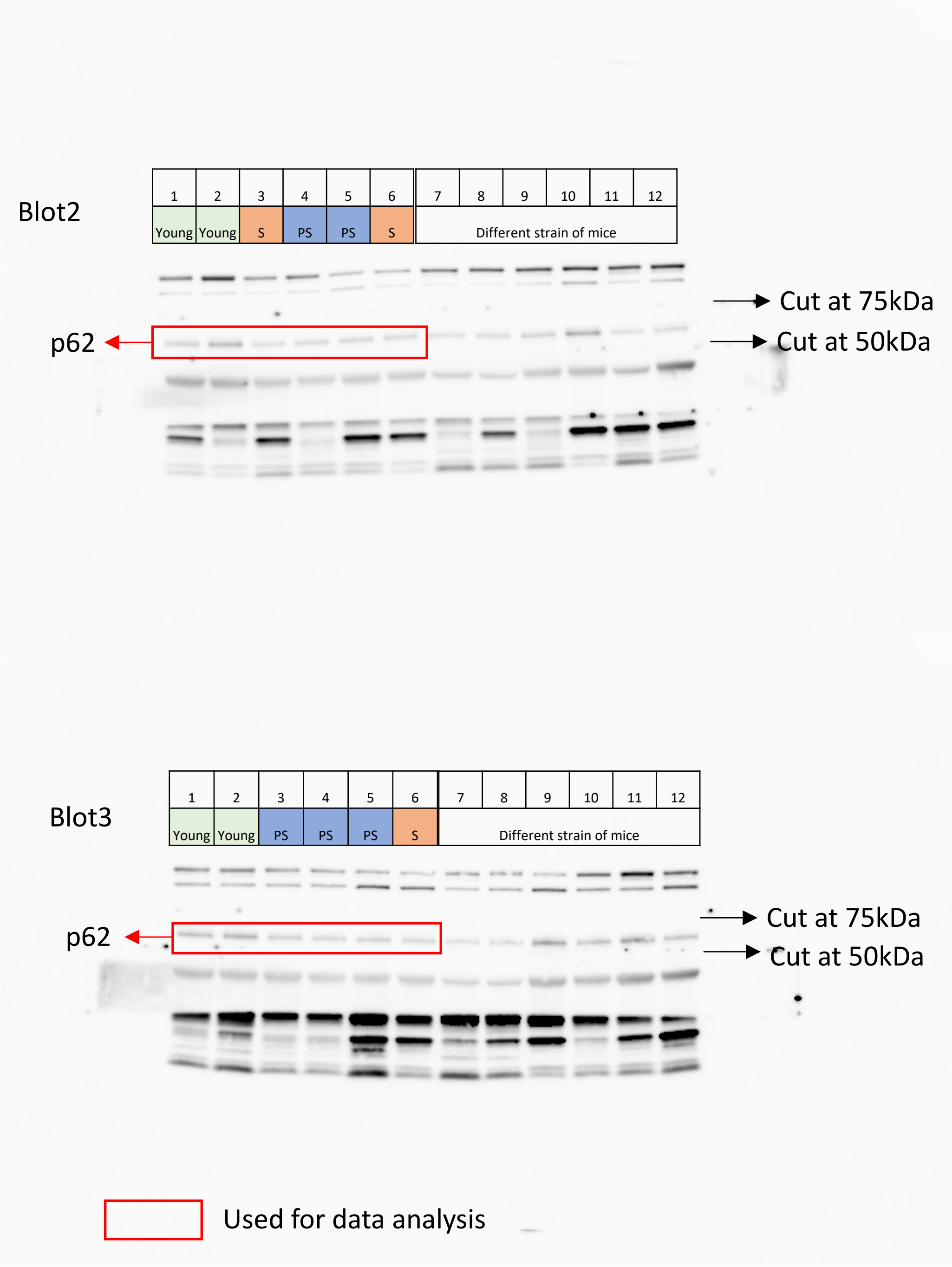

Full unedited blots for Figure 7C
MuRF1 blot1&2

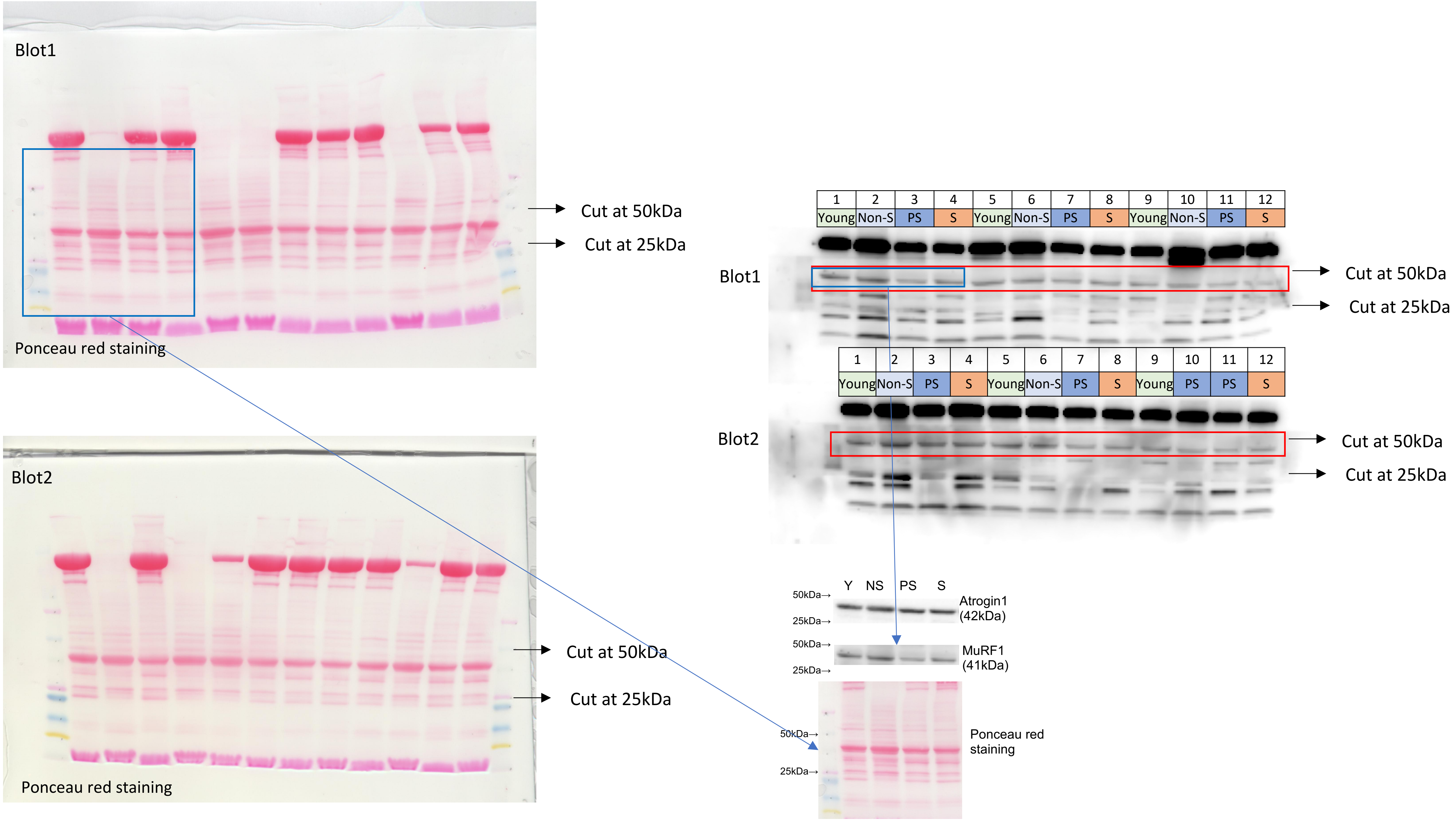

Blots are shown in Fig. 7C

Representative blots

Used for data analysis

Full unedited blots for Figure 7C
Atrogin1 blot1&2 (Stripped and reprobed after MuRF1)

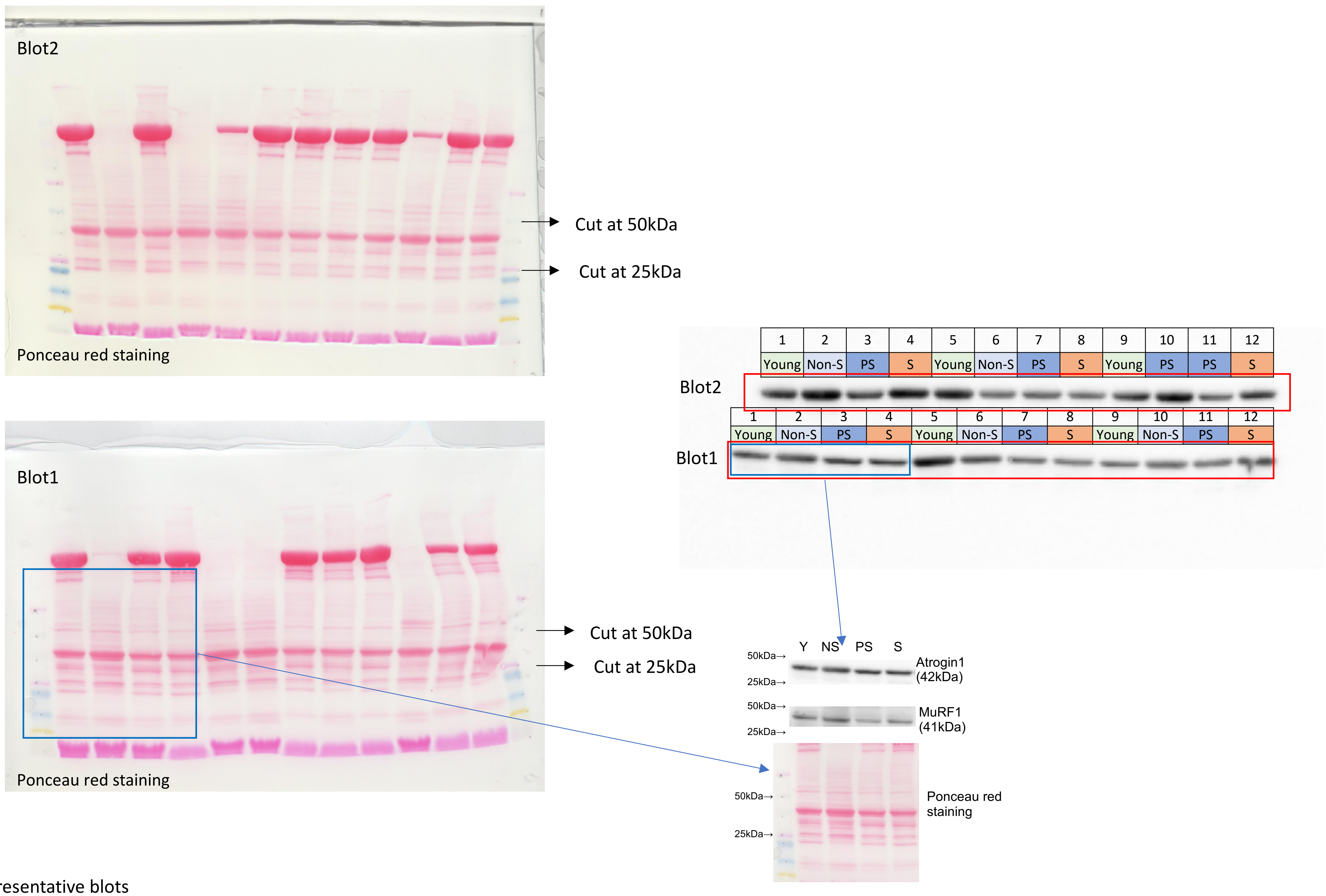

Blots are shown in Fig. 7C

Representative blots

Used for data analysis

Full unedited blots for Supplemental Fig.3I
OXPHOS complexes

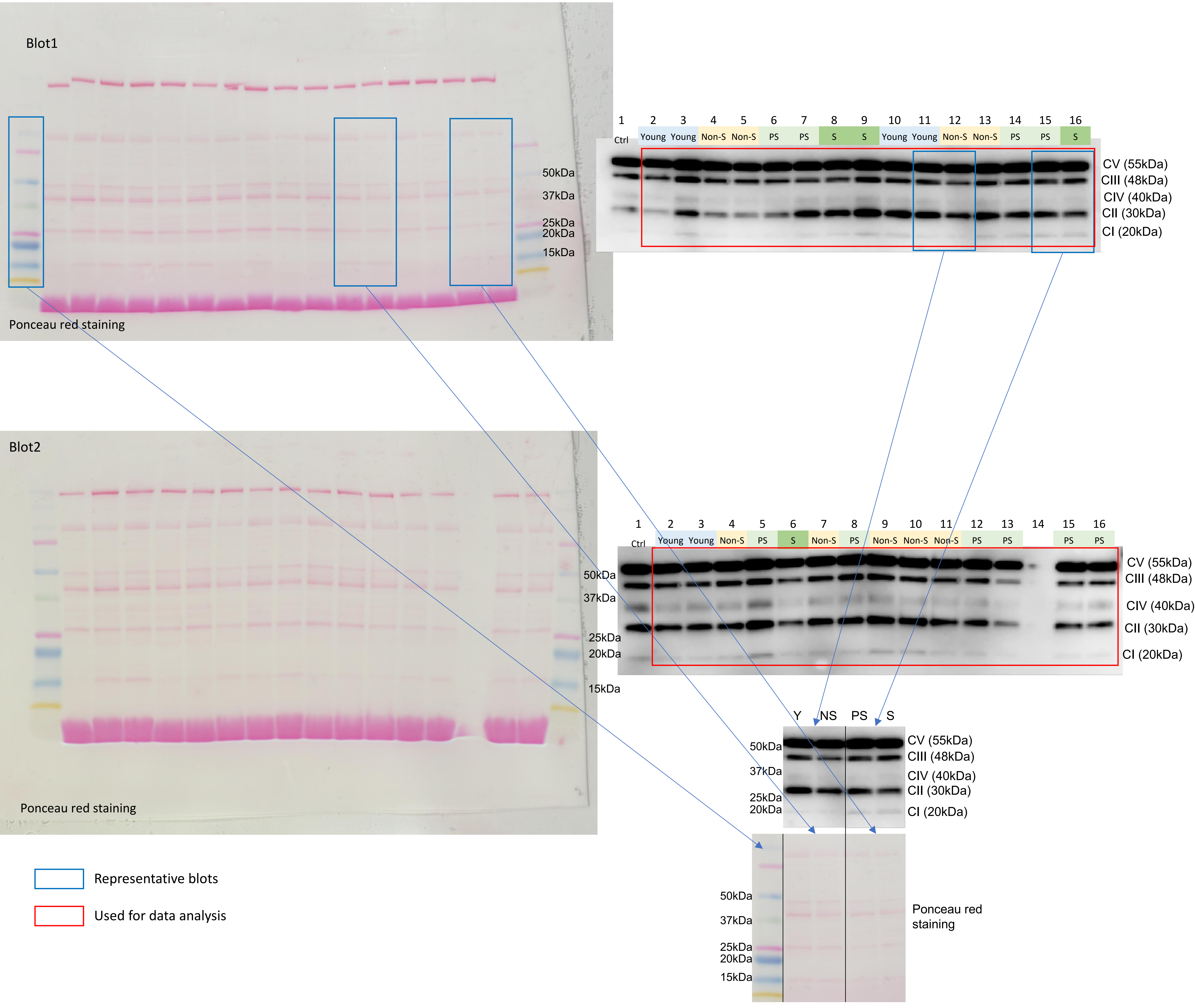

Blots are shown in Supplemental Fig. 3I

Full unedited blots for Supplemental Fig.3J
PGC1a in females

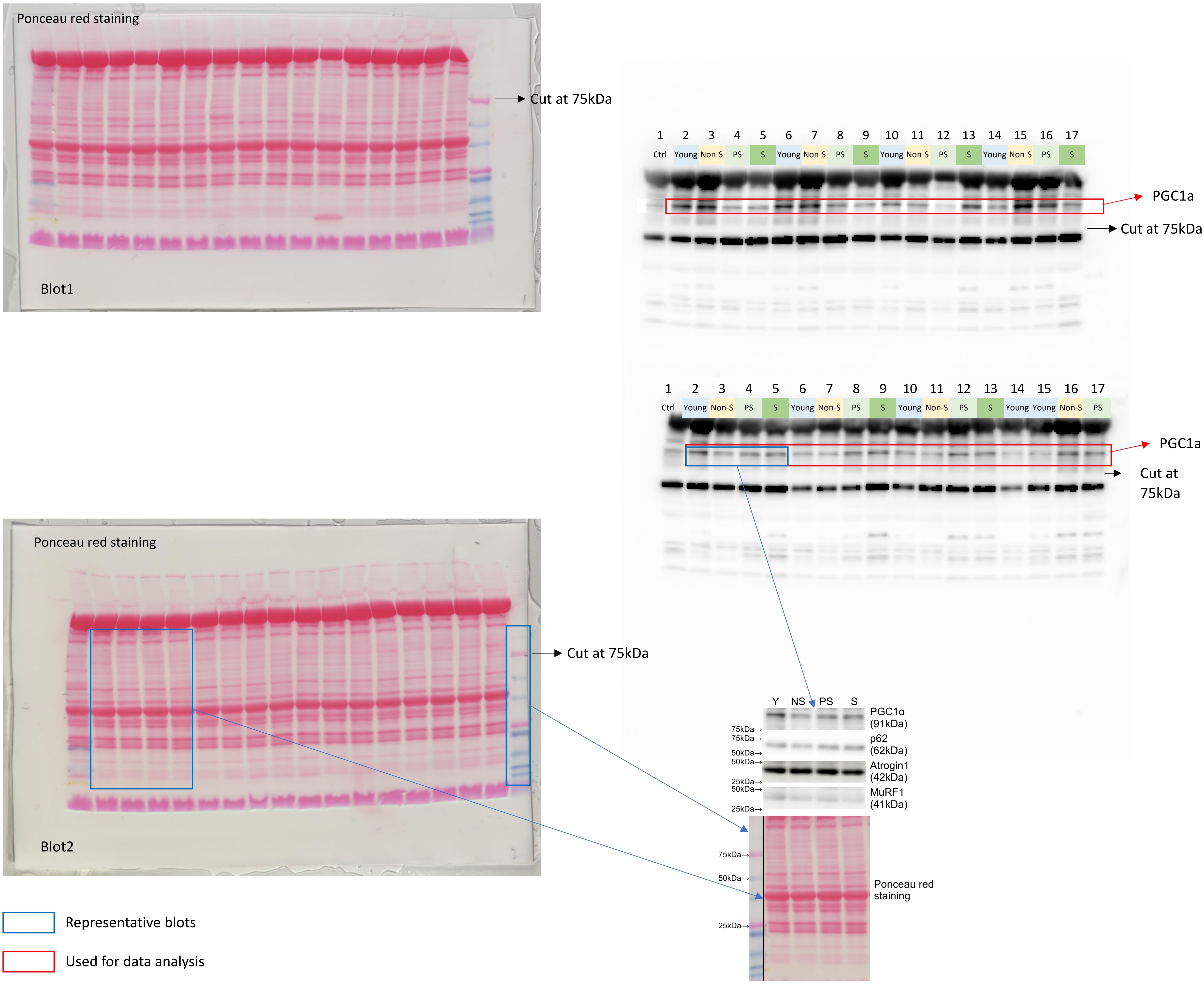

Blots are shown in Supplemental Fig. 3J

Full unedited blots for Supplemental Fig.3J
p62 in females

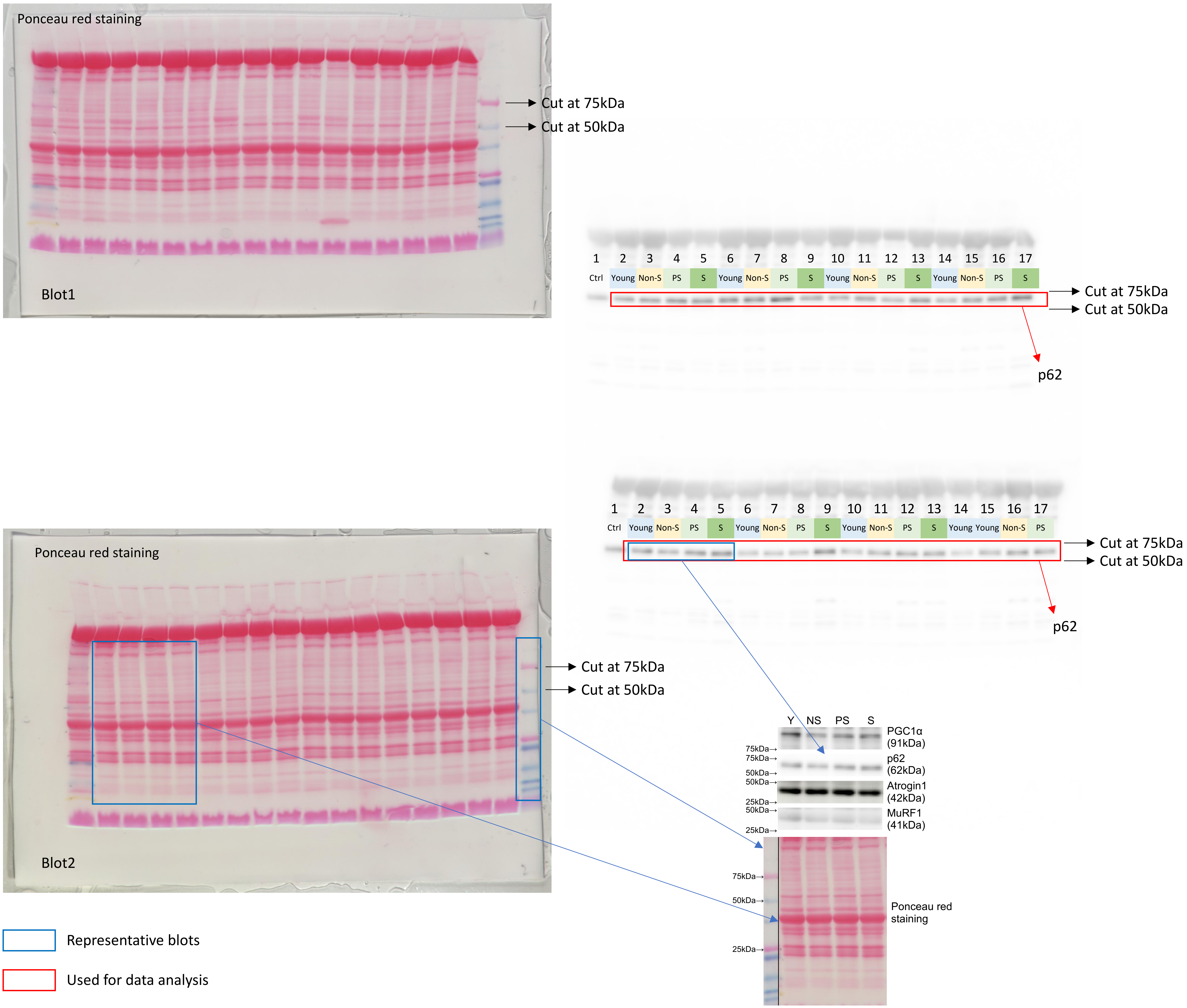

Blots are shown in Supplemental Fig. 3J

Full unedited blots for Supplemental Fig.3J
MuRF1 in females

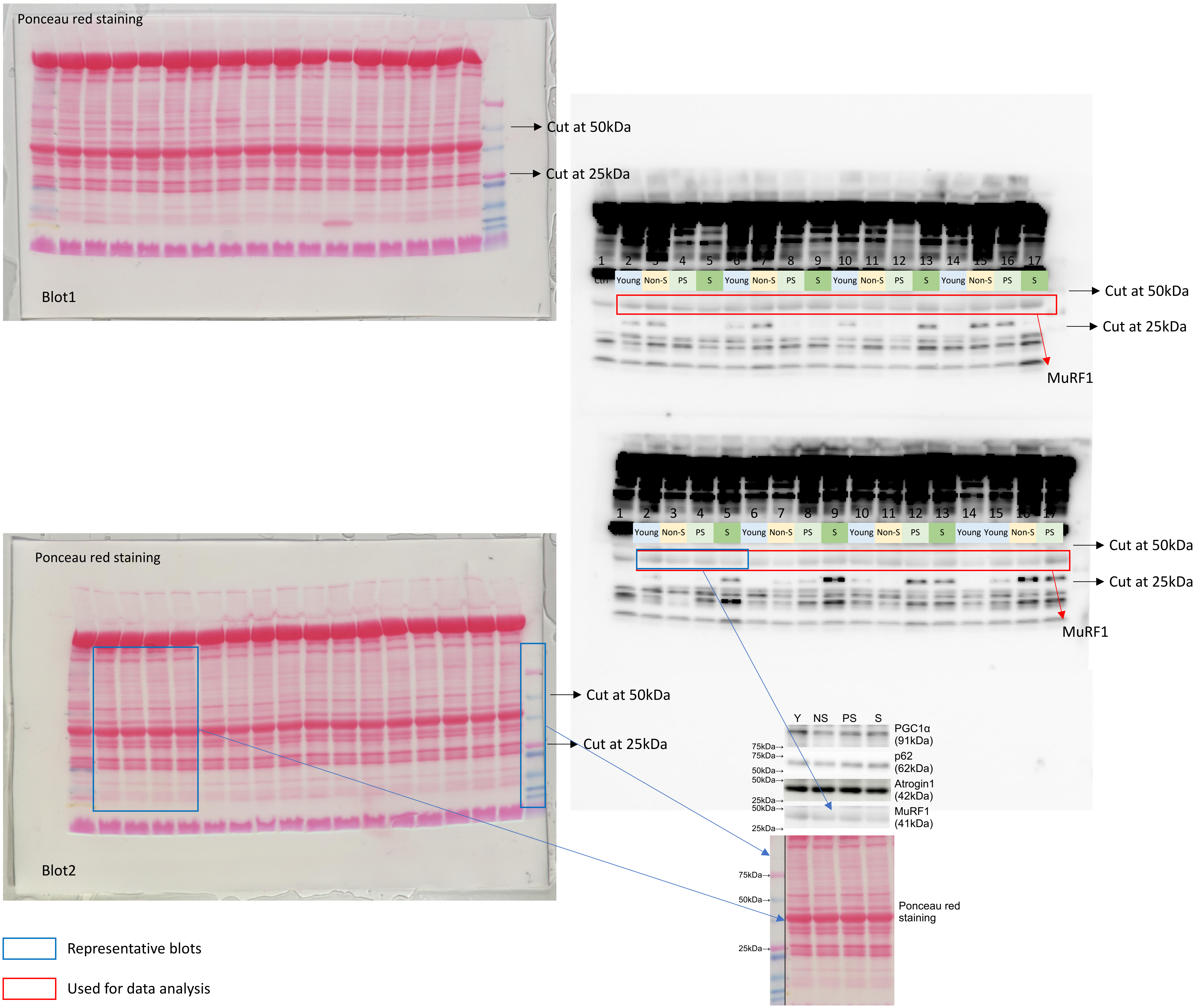

Representative blots

Used for data analysis

Blots are shown in Supplemental Fig. 3J

Full unedited blots for Supplemental Fig.3J
Atrogin1 in females
(Stripped and reprobed after MuRF1)

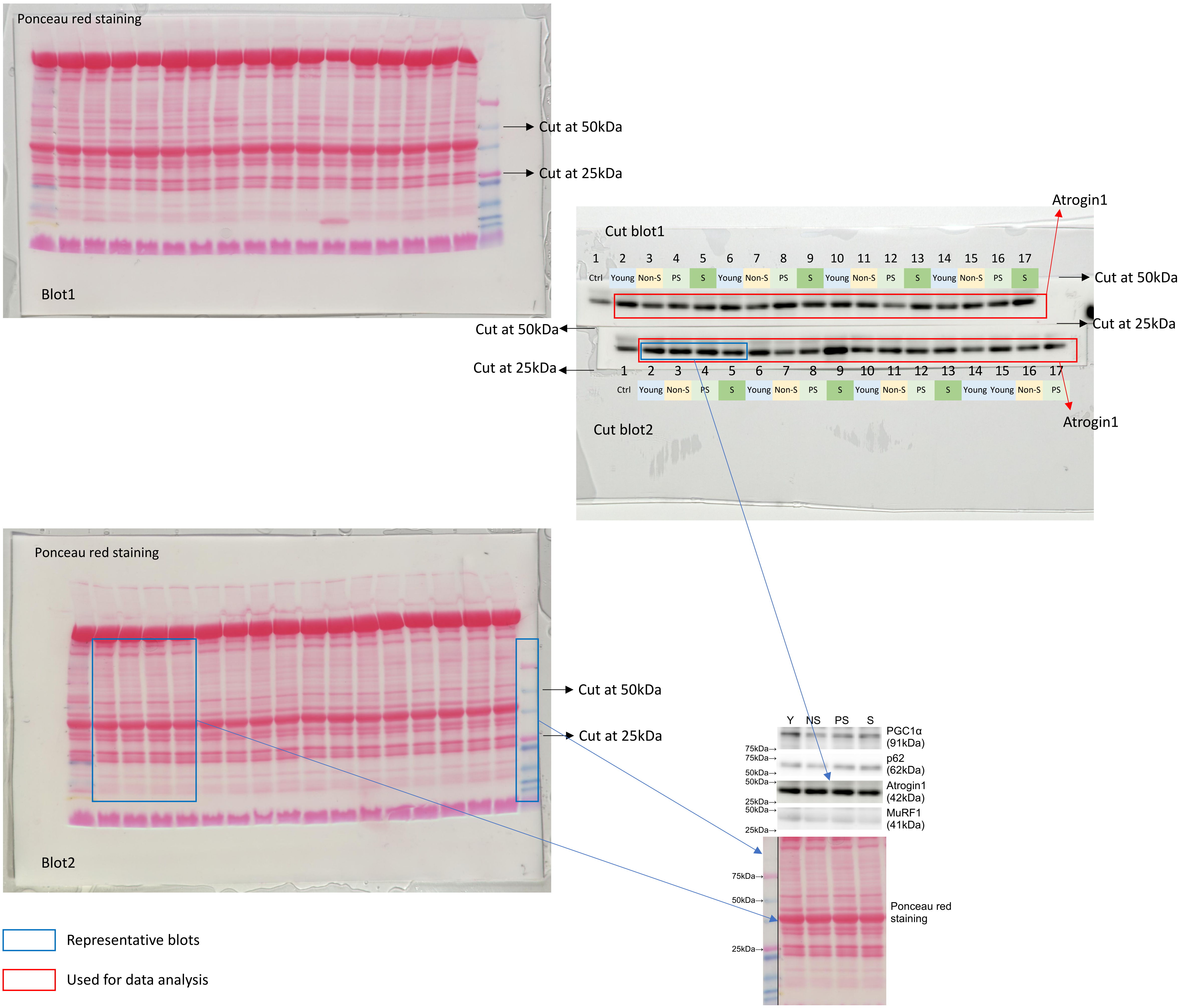

Blots are shown in Supplemental Fig. 3J
